# Human-centered industry 5.0 technologies for occupational risk reduction: a systematic review

**DOI:** 10.3389/fpubh.2026.1976362

**Published:** 2026-10-01

**Authors:** Rusber Alberto Risco-Ojeda, Cesar Moreno-Rojo, Ruben Adrián Figueroa-León, Saúl Ricardo Chuqui-Diestra, Nelver Javier Escalante-Espinoza, Juan Carlos Ponce-Ramirez, Edgar Virgilio Bedoya-Justo, Wendy Akemmy Castañeda-Rodríguez, Abel José Rodríguez-Yparraguirre, Carlos Diego Rodríguez-Yparraguirre

**Affiliations:** 1 Department of Energy, Physics and Mechanics, Faculty of Engineering, Universidad Nacional del Santa, Chimbote, Ancash, Peru; 2 Department of Agroindustry and Agronomy, Faculty of Engineering, Universidad Nacional del Santa, Chimbote, Ancash, Peru; 3 Department of Chemical Engineering, Faculty of Chemical and Metallurgical Engineering, Universidad Nacional de San Cristóbal de Huamanga, Ayacucho, Peru; 4 Department of Environmental Engineering, Faculty of Engineering and Architecture, Universidad Nacional de Moquegua, Moquegua, Peru; 5 Doctoral Program in Agro-Industrial Engineering, Specialization in Advanced Processing of Andean Grains and Tubers, Universidad Nacional del Santa, Chimbote, Ancash, Peru; 6 Doctoral Program in Science and Engineering, Graduate School, Universidad Nacional de Trujillo, Trujillo, La Libertad, Peru

**Keywords:** biomechanical overload, digital ergonomics, human-centered industry 5.0, human–robot collaboration, occupational safety, sustainable ergonomics

## Abstract

**Introduction:**

The transition to Industry 5.0 is transforming manufacturing through the integration of smart technologies, while simultaneously increasing the need to protect workers’ health and reduce occupational risks. This systematic review synthesized cross-sectoral evidence on human-centered Industry 5.0 technologies for assessing and reducing occupational risks, with a focus on ergonomic outcomes, worker-related factors, and the conditions influencing their implementation and transferability.

**Methods:**

A systematic literature search was conducted in Scopus and Web of Science following the PRISMA 2020 framework and predefined eligibility criteria based on PECO. Included studies were analyzed according to technology type, occupational risk addressed, ergonomic dimension, assessment method, reported outcomes, implementation conditions, and implications for occupational health and safety, yielding a total of 78 studies that met the inclusion criteria.

**Results:**

The reported results from individual studies included ergonomic assessment accuracies exceeding 95%, fatigue reductions of more than 50% in selected applications, and cycle-time reductions of up to 28.4%, along with improvements in task allocation, workload monitoring, and production efficiency. These values should not be interpreted as overall effects of Industry 5.0 technologies, given the heterogeneity of technologies, experimental designs, and assessment methods.

**Discussion:**

The evidence was limited by laboratory validation, a lack of longitudinal research, heterogeneous assessment methods, and insufficient real-world implementation in specific industrial sectors.

## 1 Introduction

The rapid digital transformation of industrial work environments is reshaping production systems through the integration of intelligent technologies that enhance automation, connectivity, real-time monitoring, and decision-making (1–3). Although these developments can improve operational performance, they also introduce new challenges for occupational health and safety, particularly as workers interact increasingly with automated, connected, and data-driven production systems (4, 5). Occupational risks therefore need to be addressed not only through conventional preventive measures but also through approaches capable of continuously identifying changes in physical workload, fatigue, posture, cognitive demands, and interactions between workers and technologies. In this context, the transition from Industry 4.0 toward Industry 5.0 introduces a human-centered perspective in which technological development is intended to complement human capabilities while maintaining worker well-being as a central component of industrial transformation (6, 7).

Occupational risks in manufacturing arise from the interaction of workers with tasks, equipment, organizational conditions, and physical environments. Repetitive movements, manual material handling, awkward postures, excessive physical exertion, prolonged standing, vibration, fatigue, and cognitive workload can contribute to work-related musculoskeletal disorders, reduced functional capacity, accidents, and other adverse occupational outcomes (8–11). At the same time, increasingly automated production environments may generate new forms of risk associated with human–machine interaction, technology-mediated work organization, and the need to adapt worker capabilities to rapidly changing production systems (12, 13). Consequently, effective occupational risk management requires approaches that combine accurate assessment with timely prevention and intervention. This need has strengthened interest in human-centered technologies capable of supporting occupational risk identification while preserving the active role of workers in decision-making and workplace improvement (14, 15).

Industry 5.0 provides a technological and conceptual framework for integrating advanced digital systems with human-centered occupational risk management (6, 16). Artificial intelligence can identify complex patterns in physiological, biomechanical, and behavioral data and support predictive risk assessment, whereas digital twins can simulate tasks, workstations, and human–technology interactions before changes are implemented in physical environments (17, 18). Wearable devices and the Industrial Internet of Things enable continuous acquisition of biomechanical, physiological, and contextual information, while computer vision can estimate postures, movements, and other ergonomic indicators without requiring extensive instrumentation (2, 19). Collaborative robots and exoskeletons can modify the physical demands imposed on workers, whereas virtual, augmented, and extended reality can support training, risk assessment, task simulation, and safer work design (20–23). Collectively, these technologies create opportunities to move from predominantly episodic and reactive assessments toward more continuous, data-informed, and potentially preventive approaches to occupational risk management (15, 24).

However, the contribution of these technologies to occupational risk reduction is not uniform and depends on the type of risk addressed, technological configuration, assessment methodology, level of human involvement, and context in which the intervention is implemented (25, 26). Existing studies have examined artificial intelligence, machine vision, wearable sensors, electromyography, inertial measurement units, digital twins, exoskeletons, collaborative robots, and immersive technologies across manufacturing, assembly, logistics, packaging, and human–robot collaboration environments (17, 27–31). The reported results include improvements in postural recognition, workload monitoring, fatigue assessment, task allocation, physical assistance, and ergonomic decision-making. However, the evidence is often organized around individual technologies or specific ergonomic indicators, making it difficult to determine which technological mechanisms are consistently associated with the identification, prevention, or reduction of occupational hazards in different industrial contexts (32–34). In this regard, the question arises: What technological mechanisms and contextual conditions influence the effectiveness and transferability of human-centered Industry 5.0 technologies for reducing occupational hazards in different industrial environments?

This fragmentation makes it difficult to understand how human-centered Industry 5.0 technologies reduce occupational risk, due to discrepancies in the hazards addressed, their effectiveness, and implementation requirements. Furthermore, evaluative heterogeneity, laboratory testing, and a scarcity of longitudinal analyses prevent the comparison of tools and the definition of transferable criteria (26, 32). Therefore, a systematic review is justified to classify these devices as monitoring or preventive intervention tools, since the integration of this evidence is crucial for sectors with demanding physical environments, such as seafood processing, where factors such as cold, humidity and repetitive loads generate complex risks that can be mitigated through technological solutions validated in other industries, and where technical pathways for worker well-being, from the point of view of ergonomics and occupational health, have not yet been established (33, 34).

Accordingly, this systematic review aims to critically synthesize the scientific evidence on the application of human-centered Industry 5.0 technologies for occupational risk assessment, prevention, and reduction across industrial sectors. Specifically, the review examines (i) the technologies and occupational risks addressed, (ii) the methods used to assess their effectiveness, (iii) the reported outcomes related to worker health, safety, ergonomic exposure, and workload, and (iv) the technological and contextual conditions influencing their implementation and transferability to demanding industrial environments. By integrating evidence across technological domains and occupational contexts, this review seeks to clarify the current contribution of Industry 5.0 to human-centered occupational risk management and identify priorities for future research and field-based implementation.

## 2 Methods

### 2.1 Study design

This study was designed as a cross-sector systematic review and reported in accordance with the 2020 PRISMA (Preferred Reporting Items for Systematic Reviews and Meta-Analyses) statement (35). The review synthesized empirical evidence on human-centered Industry 5.0 technologies that support the assessment, prevention, monitoring, and reduction of occupational hazards in industrial environments related to manufacturing, assembly, logistics, human-robot collaboration, and other sectors. Rather than limiting the evidence to a specific sector, the study adopted a cross-sector perspective to identify technological solutions and intervention mechanisms that address physical, biomechanical, physiological, cognitive, and task-related occupational hazards.

The synthesis emphasized experimentally reported quantitative outcomes, including ergonomic exposure, physical workload, fatigue, cognitive workload, worker safety, human-technology interaction, and technological effectiveness. Secondary operational results were considered when they were directly associated with occupational risk management, providing an evidence-based basis for assessing the applicability of human-centric Industry 5.0 technologies to demanding industrial environments. The protocol for this systematic review was not prospectively registered in PROSPERO or another publicly accessible systematic-review registry. The review was conducted according to the predefined methodological framework described in this manuscript and reported in accordance with the PRISMA 2020 statement.

### 2.2 PECO framework

The research question and eligibility scope were structured using the PECO framework, defining the population, technological exposure or intervention, comparator conditions, and relevant outcomes (36). The framework was designed to capture cross-sectoral evidence from industrial environments, with particular emphasis on technologies addressing occupational risks and supporting worker health and safety. This approach enabled the systematic identification and comparison of technological applications, risk assessment methods, intervention mechanisms, and reported outcomes across different industrial contexts, while allowing their potential transferability to demanding occupational environments such as seafood processing to be considered. The question was posed as follows: Among industrial workers (P), how do human-centered Industry 5.0 technologies (E), compared with conventional assessment or working conditions (C), affect the identification, prevention, and reduction of occupational hazards, including ergonomic, physical, cognitive, and physiological outcomes (O)? The operational definitions adopted for each PECO component are presented in Table 1.

**Table 1:** PECO framework used to define the scope of the systematic review.

Component	Definition	Operational criteria
Population (P)	Industrial workers	Workers involved in manufacturing, assembly, logistics, packaging, human–robot collaboration, and related production activities
Exposure (E)	Human-centered Industry 5.0 technologies	AI, computer vision, wearable sensors, exoskeletons, cobots, digital twins, XR, smart monitoring, and related technologies
Comparator (C)	Conventional or alternative conditions	Conventional assessment methods, baseline measurements, unsupported work conditions, control conditions, or alternative technological configurations
Outcomes (O)	Occupational health, safety, ergonomic, and human-centered outcomes	Ergonomic risk, physical/cognitive workload, fatigue, physiological responses, safety, acceptance, productivity, and operational resilience

AI, artificial intelligence; XR, extended reality; cobots, collaborative robots.

For this review, Human-Centered Industry 5.0 (HC-I5.0) was operationally defined as the application of intelligent, digital, and cyber-physical technologies in industrial environments with an explicit or demonstrable purpose of supporting, augmenting, protecting, or adapting work to human capabilities, needs, safety, well-being, or decision-making. Accordingly, the presence of an advanced technology alone was not considered sufficient to classify a study as HC-I5.0. A study was considered relevant when its technological application incorporated at least one human-centered dimension, including human–machine collaboration, worker assistance or physical augmentation, ergonomic or biomechanical assessment, occupational safety, worker health and well-being, human-centered decision support, or adaptive interaction between workers and intelligent industrial systems. Studies focused exclusively on autonomous production, machine optimization, process automation, or conventional Industry 4.0 applications without a demonstrable human-centered component were excluded.

### 2.3 Search strategy

A systematic literature search was conducted in Scopus and Web of Science (WoS) to identify studies addressing the application of digital and biomechanical technologies to human-centered ergonomics in industrial settings. The search strategy combined terms related to ergonomic risks, human factors, musculoskeletal disorders, digital technologies, artificial intelligence, wearable sensors, exoskeletons, collaborative robots, digital twins, extended reality, and smart manufacturing. The search was intentionally designed with broad industry terms rather than restricting the query to a single sector, enabling the identification of cross-sectoral evidence with potential transferability to less explored contexts. Boolean operators (AND/OR) and truncation were applied to capture variations in terminology. To maximize the sensitivity and reproducibility of the search, equivalent search strings were adapted to the indexing requirements of each database, and comprehensive Boolean strategies were implemented.

The search strategy was intentionally designed to maximize retrieval sensitivity rather than to require the explicit presence of the term “Industry 5.0” in every retrieved record. Accordingly, broad technological terms such as artificial intelligence, machine learning, IoT, computer vision, wearable sensors, exoskeletons, collaborative robots, digital twins, and extended reality were included as enabling technologies potentially supporting human-centered industrial applications. However, the presence of any of these technologies was not, by itself, considered evidence of HC-I5.0 relevance. Conceptual relevance was subsequently determined during title/abstract and full-text screening according to the operational definition and eligibility criteria described above. Studies were therefore retained only when the technological application demonstrated a clear relationship with human-centered occupational assessment, prevention, monitoring, assistance, safety, well-being, or human–technology interaction.

Scopus and Web of Science (WoS) were selected because of their multidisciplinary coverage of occupational health and safety, ergonomics, human factors, engineering, artificial intelligence, robotics, and digital technologies relevant to human-centered Industry 5.0. Their complementary indexing and citation coverage supported the systematic identification of cross-sectoral empirical evidence while maintaining a reproducible and manageable search strategy. Nevertheless, the restriction to these two databases may have resulted in the omission of relevant studies indexed in specialized or regional databases.

Scopus: (ergonomic* OR “human factors” OR “occupational ergonomics” OR “work-place ergonomics” OR “ergonomic assessment” OR “ergonomic risk assessment” OR “ergonomic risk*” OR “work-related musculoskeletal disorder*” OR WMSD* OR MSD*) AND (“Industry 5.0” OR “digital technolog*” OR “artificial intelligence” OR AI OR “machine learning” OR “deep learning” OR “digital twin*” OR “human digital twin*” OR “computer vision” OR “wearable sensor*” OR wearable* OR “smart PPE” OR exoskeleton* OR cobot* OR “collaborative robot*” OR “industrial internet of things” OR IIoT OR “internet of things” OR IoT OR “augmented reality” OR “virtual reality” OR “mixed reality” OR XR OR “edge computing”) AND (manufactur* OR “smart manufacturing” OR “intelligent manufacturing” OR “digital manufacturing” OR “advanced manufacturing” OR “food manufacturing” OR “food processing” OR “seafood processing” OR “fish processing”).

Web of science: (ergonomic* OR “human factors” OR “occupational ergonomics” OR “ergonomic risk assessment” OR “work-related musculoskeletal disorders”) AND (“digital technolog*” OR “artificial intelligence” OR “machine learning” OR “digital twin*” OR “computer vision” OR “wearable sensor*” OR exoskeleton* OR cobot* OR “industrial internet of things” OR IIoT OR “augmented reality” OR “virtual reality”) AND (manufactur* OR “food manufacturing” OR “food processing” OR “seafood processing” OR “fish processing” OR “smart manufacturing”).

### 2.4 Eligibility criteria

Studies were considered eligible when they reported empirical research evaluating human-centered digital or biomechanical technologies for occupational risk assessment, prevention, monitoring, or reduction in industrial environments. Eligible publications were peer-reviewed journal articles written in English and indexed in Scopus or Web of Science, with open-access full text and sufficient methodological and empirical information to identify the technological intervention, application context, assessment approach, and principal occupational outcomes. The open-access criterion was applied to ensure full-text accessibility for independent screening, data extraction, and verification by the reviewers, rather than as an indicator of scientific quality or methodological rigor. Studies conducted in manufacturing, assembly, logistics, packaging, human–robot collaboration, and related production environments were included to support the cross-sectoral synthesis and identify evidence potentially transferable to demanding occupational contexts. Studies were excluded when they were systematic or narrative reviews, conference papers, editorials, book chapters, purely conceptual or theoretical works, unrelated to occupational health, safety, or ergonomics, or lacked sufficient empirical evidence to evaluate the reported technological application or occupational outcomes.

### 2.5 Selection process and data extraction

The study selection process was conducted according to the Preferred Reporting Items for Systematic Reviews and Meta-Analyses (PRISMA 2020) guidelines. The initial search retrieved 2,382 records, comprising 1970 records from Scopus and 412 from Web of Science (WoS). In Scopus, the application of the publication-year filter (2021–2026) reduced the records to 1,321, excluding 649 documents; restricting the results to journal articles reduced the dataset to 557 records, excluding a further 764 documents, which constituted the Scopus records entering the identification stage. In WoS, the initial 412 records were reduced to 352 after applying the 2021–2026 filter, excluding 60 records; the document-type filter reduced the dataset to 289 articles, excluding 63 records. The filtered datasets were subsequently combined, resulting in 846 records. These records were imported and harmonized using the bibliometrix package in RStudio, where 236 duplicate records were identified and removed, yielding 610 unique records for the subsequent PRISMA screening stage. This sequential filtering process ensured that the evidence base was restricted to recent, peer-reviewed, available for detailed methodological and thematic assessment.

During the title-and-abstract screening stage, the 610 records were independently assessed by two reviewers (C. M.-R and C. D. R.-Y) according to the predefined eligibility criteria. Inter-reviewer agreement was evaluated using Cohen’s kappa coefficient (κ) based on a 2×2 contingency matrix. The reviewers independently agreed on the inclusion of 120 records and the exclusion of 452 records, while disagreements occurred for 38 records, comprising 18 records classified as included by C. M.-R but excluded by C. D. R.-Y and 20 records classified as excluded by C. M.-R but included by C. D. R.-Y. The observed agreement (Pₒ) was 0.938, whereas the expected agreement by chance (Pₑ) was 0.648, resulting in a Cohen’s kappa coefficient of κ=0.82 . This value indicates a high level of inter-reviewer agreement, supporting the consistency and reliability of the screening procedure. Disagreements ( n=38 ) were first discussed between the two reviewers; cases that remained unresolved were adjudicated by a third, independent reviewer, A. J. R.-Y, whose decision was final. The screening and eligibility assessment subsequently followed the PRISMA sequence (Figure 1).^1^ From the 610 unique records entering the screening stage, 472 records were excluded following examination of their titles and abstracts because they did not sufficiently address human-centered ergonomics, Industry 4.0/5.0 technologies, occupational applications, or the defined outcome domains. The remaining 138 records were sought for full-text assessment; 29 records were excluded because the full text could not be retrieved, resulting in 109 articles available for detailed eligibility assessment. Full-text examination led to the exclusion of 31 studies because their findings were predominantly descriptive, they did not provide sufficient experimental procedures or empirical evidence, or their thematic focus was substantially distant from the objectives of the review. Following full-text eligibility assessment, data were extracted from the included studies using a standardized extraction matrix developed specifically for this review. Data extraction was performed by two reviewers (C. M.-R. and C. D. R.-Y.), who independently assessed the eligible studies and recorded the predefined variables. The extracted information included bibliographic characteristics (author, publication year, and country), industrial sector and application context, study design and participant characteristics, technology category and specific technological implementation, human-centered function, occupational risk or ergonomic domain addressed, assessment method or measurement instrument, comparator or baseline condition when available, principal quantitative outcomes, reported technological performance, implementation conditions, and relevant limitations. The extraction records were subsequently compared by the two reviewers to identify inconsistencies. Discrepancies were discussed to reach consensus and, when consensus could not be achieved, were adjudicated by a third independent reviewer (A. J. R.-Y.), whose decision was final. Missing or insufficiently reported information was recorded as “not reported” and was not inferred from other sources.

**Figure 1:**
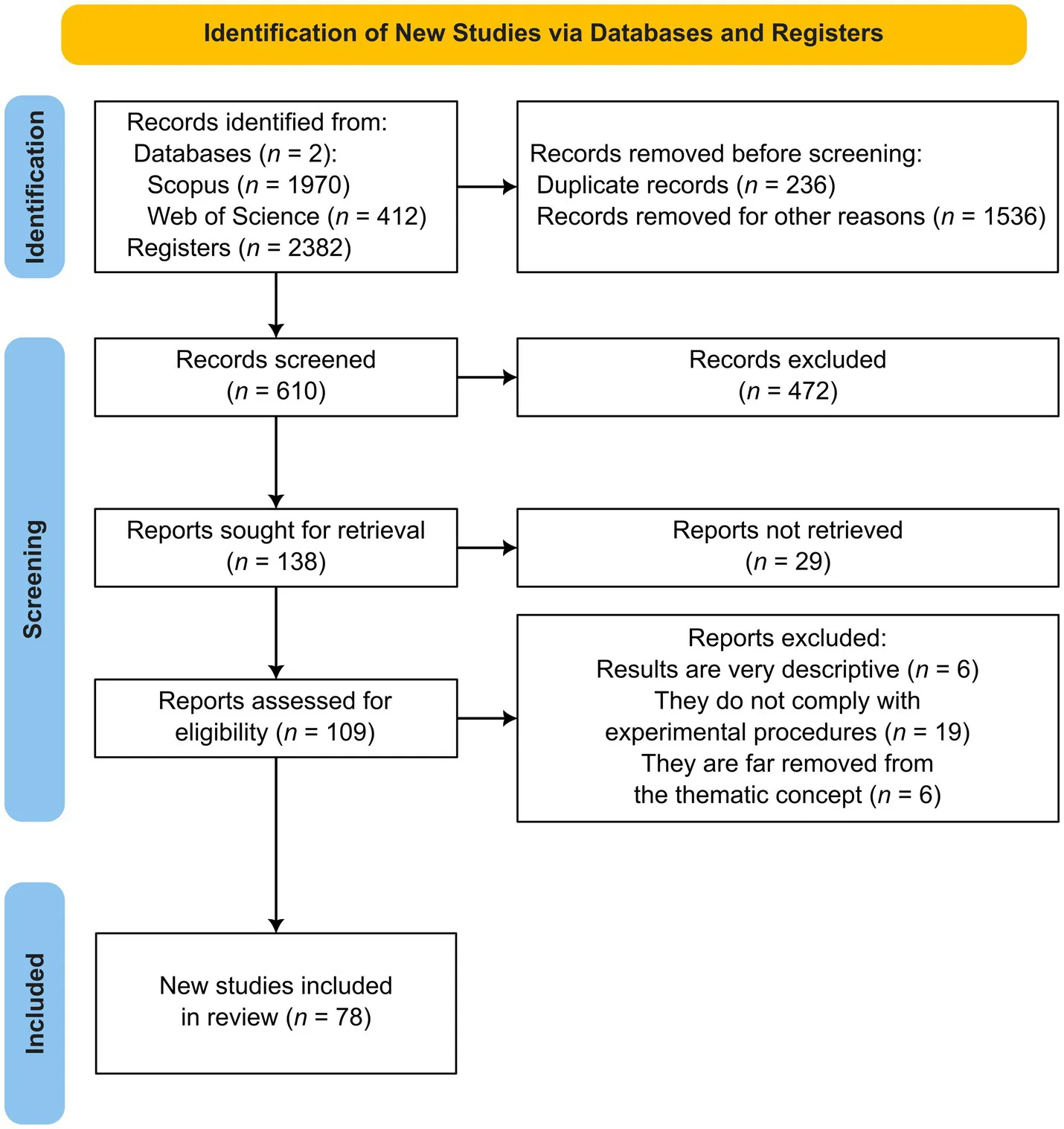
PRISMA 2020 flow diagram illustrating the systematic identification, screening, eligibility assessment, and inclusion of studies.

### 2.6 Quality and risk-of-bias assessment

The methodological quality and risk of bias of the included studies were independently assessed by two reviewers using a structured five-domain matrix developed specifically for this systematic review. The framework was based on predefined methodological questions relevant to technology-oriented occupational health and safety research and accommodated heterogeneous evidence involving artificial intelligence, computer vision, wearable sensors, exoskeletons, collaborative robots, digital twins, and virtual or augmented reality. Five methodological domains were assessed: D1, selection of participants or scenarios; D2, validity of technological and occupational-risk measurements; D3, experimental or application setting; D4, reporting completeness and transparency; and D5, potential confounding and other sources of bias. Each domain was evaluated through specific methodological questions.

For each study, reviewers independently answered the predefined questions within the five domains and assigned a qualitative rating of low risk, some concerns, or high risk according to the available methodological information reported. Low risk indicated adequate fulfillment of the evaluated criteria; some concerns indicated partial reporting, methodological limitations, or insufficient information; and high risk indicated substantial limitations that could affect the credibility of findings. Domain ratings were recorded in the assessment matrix, and the overall risk-of-bias classification was determined from the highest-risk domain identified across the five domains. Disagreements were discussed and resolved by consensus before the final classification was established.

Methodological quality and risk of bias were considered related but distinct components of the assessment. Methodological quality referred to the adequacy, completeness, and transparency of the study design and reporting, whereas risk of bias referred to the potential for systematic methodological limitations to distort the reported findings. Accordingly, the five-domain matrix was used to evaluate methodological characteristics and their implications for potential bias, while the final classification represented the reviewers’ judgment of the potential risk that these limitations could influence the study results. Domain ratings were recorded in the assessment matrix, and the overall risk-of-bias classification was determined from the highest-risk domain identified across the five domains. Disagreements were discussed and resolved by consensus before the final classification was established. The complete assessment is presented in Figure 2.

**Figure 2:**
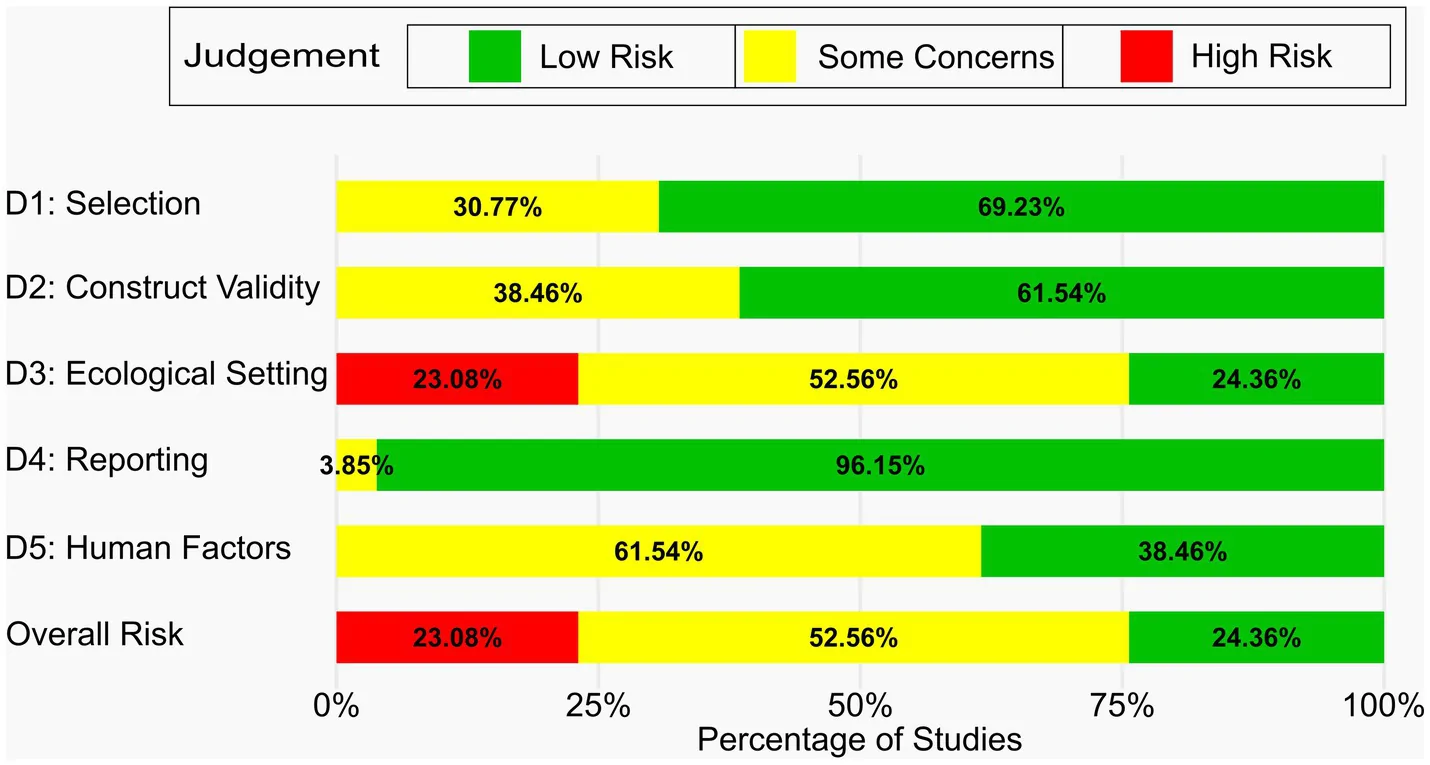
Risk of bias and methodological quality assessment ( N=78 ). Five domains were evaluated: D1 (Selection and scenario representativeness), D2 (Construct validity and measurement reliability), D3 (Ecological setting and shop-floor applicability), D4 (Reporting adequacy and transparency), and D5 (Human factors and confounding control). Overall risk was calculated using the domain-level worst-case rule. The overall high-risk proportion ( 23.08%,n=18 ) matches D3 exactly, as no high-risk scores were observed in Domains 1, 2, 4, or 5.

The risk-of-bias assessment was incorporated into the interpretation of evidence synthesis rather than as a descriptive result. Studies classified as low risk were considered to provide stronger methodological support for their findings, whereas studies presenting some concerns or high risk were interpreted more cautiously. Domain-level results were examined to identify recurring limitations in participant or scenario selection, measurement validity, experimental settings, reporting, and confounding. These findings informed comparisons across technologies and occupational-risk outcomes, particularly where methodological weaknesses could influence reported effects. Thus, the assessment provided a basis for judging the robustness of the synthesized evidence and qualifying conclusions when findings were supported by studies with substantial concerns or high risk of bias.

### 2.7 Data synthesis and analysis

Given the substantial heterogeneity in technologies, occupational risks, assessment methods, and outcome metrics across the included studies, a quantitative meta-analysis was not conducted. Instead, findings were synthesized narratively and organized according to technological domain, occupational risk addressed, intervention mechanism, and reported worker-related outcomes. Studies were grouped and tabulated according to technology type, application context, assessment approach, occupational risk, and reported outcomes to identify convergent patterns, quantitative ranges, implementation conditions, and evidence gaps across industrial sectors. The synthesis distinguished technologies primarily supporting risk detection and monitoring from those enabling preventive or corrective interventions. No sensitivity analyses were performed because the review did not involve pooled effect estimates or statistical meta-analysis.

## 3 Results

### 3.1 Study selection and bibliographic characteristics

The included studies covered a broad range of industrial sectors, confirming the cross-sectoral nature of Human-Centered Industry 5.0 research for sustainable ergonomics and operational resilience (37). Manufacturing represented the predominant application context, particularly through automotive, garment, assembly, machining, logistics, and discrete-production environments, where collaborative robots, computer vision, wearable devices, digital twins, augmented and virtual reality, and artificial intelligence were applied to address ergonomic and operational challenges (38, 39). Additional evidence was identified in handcrafted manufacturing, warehouse operations, plastics production, furniture assembly, and other industrial activities characterized by repetitive tasks, manual material handling, variable workloads, and human–robot interaction. This sectoral diversity is relevant because the ergonomic challenges addressed in these contexts (physical workload, biomechanical fatigue, cognitive workload, task variability, safety, and adaptive task allocation) can also be extrapolated to seafood processing activities.

Figure 3 shows a sustained increase in scientific output, from 5 publications in 2021 to 19 in 2026, with a significant trend ( R2=0.9657;p<0.001 ). Topics evolved from Digital Twin to immersive technologies, artificial intelligence, ergonomic assessment, human-robot collaboration, and wearable sensors. “Occupational Risk and Ergonomics” registered 106 occurrences, followed by Human-Robot Collaboration (40) and Human-Centered Systems (41). Italy led the output with 21 publications, followed by China (9) and the United States (7). Applied Sciences achieved the highest local H-index (6), while Bradford identified 4 sources in the central region, confirming a specialized concentration of the literature.

**Figure 3:**
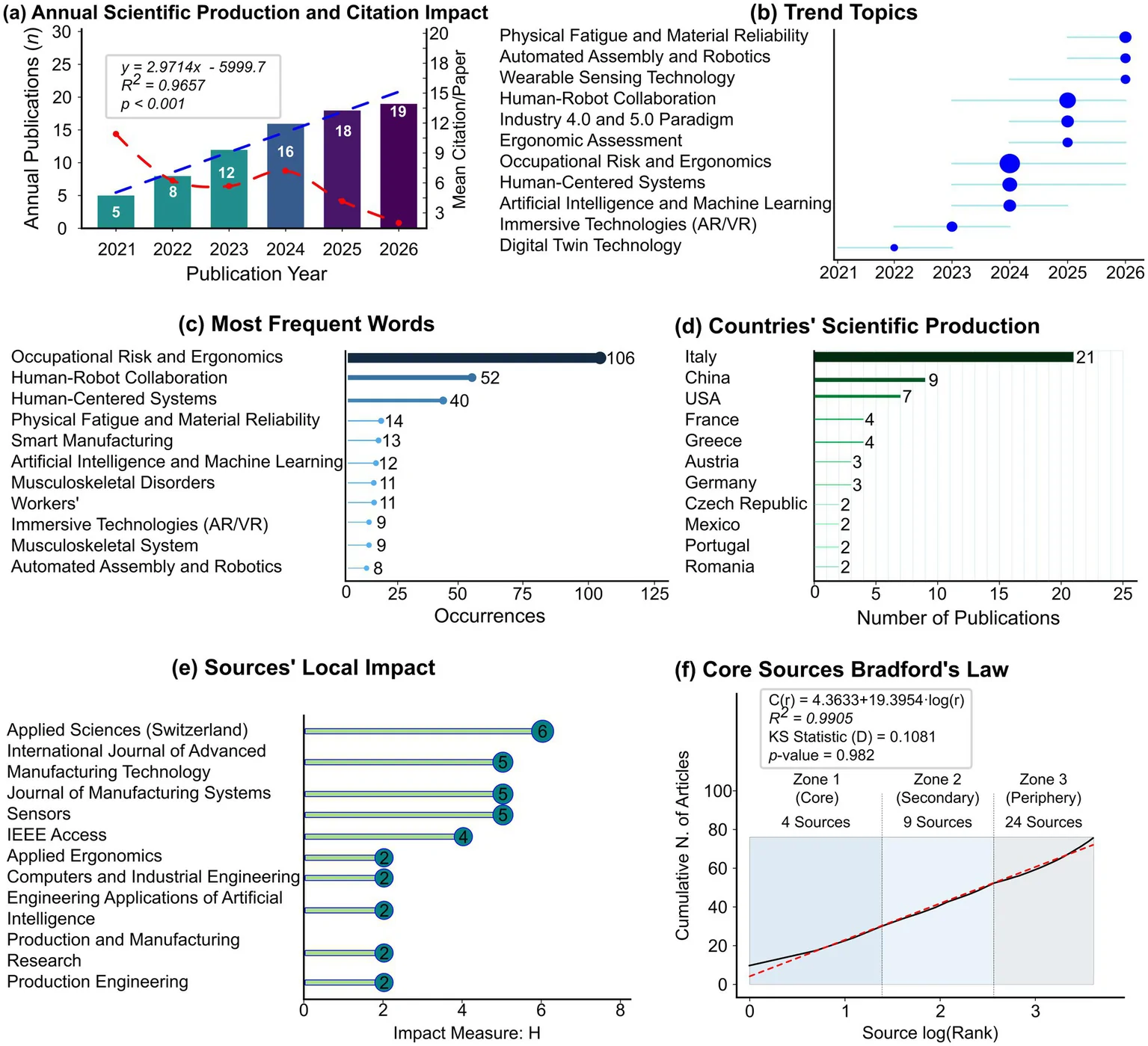
Bibliometric analysis of global scientific production on human-centered Industry 5.0, sustainable ergonomics, and operational resilience. **(a)** Annual evolution of scientific publications (bars) and mean citation impact per paper (line) between 2021 and 2026. **(b)** Temporal distribution and growth of trend topics within the research field. **(c)** Frequency of the most common keywords. **(d)** Scientific production distributed by countries of origin. **(e)** Ranking of local impact of sources based on the H-index. **(f)** Distribution of core scientific journals applying Bradford’s Law across Zones 1, 2, and 3.

Studies demonstrate a progressive integration of human-centered technologies in manufacturing environments, especially where it is necessary to simultaneously address ergonomic risks, worker well-being, and operational performance. This trend is reflected in the diversity of technological approaches identified, including wearable sensors, ma-chine vision, artificial intelligence, collaborative robots, exoskeletons, virtual and augmented reality, and digital twins. Their application in manufacturing, assembly, textiles, construction, automotive, and other production sectors indicates that ergonomic assessment is increasingly dependent on technologies capable of continuously monitoring physical and cognitive demands, predicting fatigue or risk, and adapting human-robot interactions. This technological orientation is relevant to sustainable manufacturing, as it links worker protection and well-being with safer, more efficient, and adaptable production systems. The main characteristics of the included studies are summarized in Table 2, which serves as the basis for the subsequent analysis of technological applications and their contributions related to ergonomics and sustainability.

**Table 2:** Characteristics of the included studies ( N=78 ).

Ref.	Country	Productive sector	Technology	Ergonomic focus	Subject area
Wang et al. (39)	China	Manufacturing	Digital twin	Work ergonomics	Engineering
Salami et al. (41)	Italy	Manufacturing	Cobots	Physical workload	Engineering
You et al. (88)	United Kingdom	Manufacturing	Human digital twin	Physical fatigue	Computer Science
Pistolesi et al. (85)	Italy	Manufacturing	Smartwatch + LiDAR	Postural risk	Computer Science
Simeone et al. (104)	Italy	Manufacturing	Cyber-physical system	Risk assessment	Engineering
Morandini et al. (42)	Italy	Automotive	Exoskeleton + cobot	Multidimensional	Engineering
Morillo et al. (75)	Colombia	Pharmaceutical	Multimodal sensing	Multidimensional	Engineering
Agostinelli et al. (62)	Italy	Manufacturing	Computer vision	Postural risk	Engineering
Bonello et al. (99)	Malta	Manufacturing	Cobots	Physical/cognitive	Engineering
Baklouti et al. (87)	France	Manufacturing	IMU wearable	MSD risk	Computer Science
Generosi et al. (63)	Italy	Manufacturing	Computer vision	Ergonomic risk	Engineering
Mao et al. (43)	China	Automotive	Augmented reality	Physical fatigue	Engineering
Harith et al. (44)	Malaysia	Agriculture	Exoskeleton	Muscle activity	Engineering
Papoutsakis et al. (40)	Greece	Automotive	Computer vision	Postural risk	Computer Science
Chand et al. (89)	New Zealand	Manufacturing	Human digital twin	Physical fatigue	Engineering
Calzavara et al. (45)	Italy	Manufacturing	Cobots	Physical workload	Engineering
Pluchino et al. (51)	Italy	Manufacturing	Cobots + eye tracking	Mental workload	Engineering
Ciccarelli et al. (46)	Italy	Manufacturing	Cobots + VR	Physical workload	Engineering
Muntean et al. (52)	Romania	Manufacturing	IMU + AI	Upper limb	Engineering
Flores-García et al. (76)	Sweden	Logistics	AI vision	Human factors	Engineering
Domínguez-Ruiz et al. (90)	Mexico	Manufacturing	AI + digital twin	Fatigue risk	Engineering
Zhang et al. (72)	China	Manufacturing	Computer vision + AI	Fatigue risk	Computer Science
Xuan et al. (47)	China	Manufacturing	Digital twin	Workload	Engineering
Lukodono & Lin (53)	China	Manufacturing	EMG + ML	Muscle activity	Computer Science
Tran et al. (105)	Hungary	Manufacturing	Kinect sensor	Work performance	Engineering
Borghi et al. (54)	Italy	Manufacturing	Multimodal sensing	Psychophysiological stress	Engineering
Panchetti et al. (64)	Italy	Manufacturing	Cobots	Cognitive workload	Engineering
Malega et al. (106)	Slovakia	Manufacturing	Augmented reality	User ergonomics	Engineering
Pawełczyk et al. (107)	Czech Republic	Manufacturing	Robot + AGV	Physical workload	Engineering
Sanna et al. (92)	Austria	Manufacturing	AR + BCI	Cognitive workload	Computer Science
Coccia et al. (48)	Italy	Manufacturing	Exoskeleton	Muscle activity	Engineering
Nacarino et al. (77)	Peru	Healthcare	Exoskeleton	Rehabilitation	Engineering
Kulaç & Kiraz (100)	Turkey	Manufacturing	Cobots	Multidimensional	Engineering
Nasirzadeh et al. (78)	Australia	Construction	Wearable sensors	Physical fatigue	Engineering
Krishnan et al. (55)	United States	Manufacturing	Multimodal sensors + ML	Hand ergonomics	Engineering
Vukicevic et al. (83)	Serbia	Manufacturing	Deep learning + computer vision	Postural risk	Computer Science
Papoutsakis et al. (69)	Greece	Automotive	Computer vision + smartwatch	Physical fatigue	Computer Science
Ahn et al. (108)	Korea	Manufacturing	Smart garments	Ergonomic design	Engineering
Tomelleri et al. (93)	Italy	Manufacturing	IoT + smart glove + computer vision	Musculoskeletal risk	Engineering
Maruyama et al. (49)	Japan	Manufacturing	Digital twin	Physical workload	Engineering
Jia et al. (84)	Germany	Manufacturing	Deep reinforcement learning	Workload	Computer Science
Donati et al. (56)	Italy	Manufacturing	ECG + machine learning	Work stress	Engineering
Khan et al. (102)	Germany	Manufacturing	Cobots + remote systems	Human factors	Engineering
Kačerová et al. (70)	Czech Republic	Automotive	VR + motion capture	Workplace ergonomics	Engineering
Keshvarparast et al. (57)	France	Manufacturing	Cobots + IMU + sEMG	Physical fatigue	Engineering
Vora et al. (91)	United States	Manufacturing	Exoskeleton	Fatigue	Engineering
Davoudi Kakhki et al. (109)	United States	Manufacturing	Exoskeleton	Fatigue	Engineering
Gervasi et al. (58)	Italy	Manufacturing	Eye-tracking	Mental workload	Engineering
Zigart et al. (50)	Austria	Manufacturing	AR/VR	Workload	Engineering
Benmessabih et al. (80)	France	Manufacturing	Graph-based AI	Fatigue	Computer Science
Tasneem & Pieters (110)	Finland	Manufacturing	Human–robot interaction (LLM)	Inspection	Computer Science
Ofori et al. (79)	United States	Construction	Exoskeleton	Discomfort	Engineering
Keshvarparast et al. (96)	Canada	Manufacturing	Cobots	Fatigue	Engineering
Mares-Castro et al. (111)	Mexico	Manufacturing	Computer vision	Posture	Computer Science
Dalle Mura & Dini (65)	Italy	Manufacturing	Cobots	Energy load	Engineering
Cruciata et al. (86)	Italy	Manufacturing	Vision Transformer	Posture	Computer Science
Su et al. (59)	China	Manufacturing	Machine learning	Posture	Computer Science
Sharotry et al. (97)	United States	Manufacturing	Digital twin	Fatigue	Computer Science
Zakeri et al. (60)	United Kingdom	Manufacturing	EEG/fNIRS	Mental workload	Neuroscience
Colim et al. (112)	Portugal	Manufacturing	Cobots	Physical load	Engineering
Martinelli et al. (66)	Italy	Manufacturing	Deep learning	Workstation design	Engineering
Matos et al. (113)	Portugal	Manufacturing	Motion capture + ML	MSD prevention	Engineering
Hijry et al. (61)	France	Manufacturing	IoT + ML	Work stress	Computer Science
Terlizzi et al. (94)	Italy	Manufacturing	Exoskeleton + sEMG	Muscle fatigue	Engineering
Liu et al. (114)	China	Manufacturing	Cobot + Fuzzy logic	Human intention	Computer Science
Xue et al. (81)	Hong Kong	Manufacturing	Safe RL	Physical fatigue	Engineering
Dimitropoulos et al. (95)	Greece	Manufacturing	AI + Wearables	Posture	Engineering
Bittencourt et al. (82)	Netherlands	Manufacturing	Digital human + ML	Workstation design	Engineering
Lin & Lukodono (73)	Taiwan	Manufacturing	Deep learning	Fatigue prevention	Computer Science
Zörrer et al. (67)	Austria	Manufacturing	HRC + visual guidance	Mental workload	Engineering
Dall’Alba and Boriero (103)	Italy	Manufacturing	Cobot + gamepad	Usability	Engineering
Agostinelli et al. (68)	Italy	Manufacturing	Computer vision	Ergonomic risk	Engineering
Dylong & Nickel (115)	Germany	Manufacturing	Virtual reality	Mental workload	Psychology
Maddipatla et al. (101)	Unites States	Manufacturing	Virtual reality + AI	Training	Engineering
Arkouli et al. (116)	Greece	Manufacturing	Axiomatic design	Task design	Engineering
Javernik et al. (98)	Slovenia	Manufacturing	Cobot + HRV	Physiological stress	Psychology
Nioata et al. (71)	Romania	Automotive	SMIS	Ergonomic risk	Engineering
Tan et al. (74)	China	Manufacturing	Computer vision	Risk assessment	Engineering

The table summarizes the characteristics of the studies included in the systematic review published between 2021 and 2026, focusing on human-centered Industry 5.0 technologies applied to ergonomic assessment, worker well-being, occupational safety, operational efficiency, and sustainable manufacturing. Light detection and ranging (LiDAR); inertial measurement unit (IMU); brain-computer interface (BCI); augmented and virtual reality (AR/VR); large language models (LLMs); electroencephalography (EEG), functional near-infrared spectroscopy (fNIRS); machine learning (ML); Musculoskeletal Disorders (MSD); internet of things (IoT); electromyography (EMG); safe reinforcement learning (RL); artificial intelligence (AI); human-robot collaboration (HRC); heart rate variability (HRV); integrated smart manufacturing systems (SMIS).

### 3.2 Risk of bias of included studies

Overall, the methodological quality of the included studies varied across the five evaluated domains. The majority of studies exhibited a low risk of bias regarding measurement validity and protocol clarity, driven by the use of validated technological instrumentation and standardized experimental setups. However, high risk of bias and some concerns were frequently observed in participant selection and sample size adequacy, as many experiments were conducted in controlled laboratory settings with limited sample sizes. A detailed breakdown of the risk of bias proportions across all evaluated domains is summarized in Figure 2, while the individual assessment for each of the 78 included studies is presented in Supplementary Table S2.

### 3.3 Human-centered industry 5.0 technologies for occupational risk assessment

Scientific evidence published between 2021 and 2026 indicates an evolution from the initial application of collaborative technologies toward more intelligent, adaptive, and continuously monitored human-centered systems for occupational risk assessment. Early research focused on collaborative robots, exoskeletons, augmented reality, digital twins, and sensor-based systems as mechanisms for identifying physical workload, supporting safer task execution, and improving human–technology interaction (41–50). Subsequent studies expanded this approach through multimodal sensing, artificial intelligence, computer vision, machine learning, and physiological monitoring, enabling the assessment of occupational risks through continuous and data-driven approaches (51–61). This evolution reflects the increasing need to identify changes in posture, physical workload, fatigue, stress, and other worker-related risk factors dynamically during task execution.

Regarding the evolution of application areas, manufacturing remains the predominant context, particularly assembly operations, where repetitive movements, manual material handling, workstation configuration, and human–robot collaboration generate substantial occupational risk exposure (41, 45, 46, 51, 55, 57, 62–68). Automotive manufacturing represents another important application area, with technologies addressing physical fatigue, collaborative robotics, machine vision, and integrated smart manufacturing systems (40, 42, 43, 69–71). Textile and apparel production also emerges as a relevant context because repetitive upper-limb movements and constrained postures contribute to persistent musculoskeletal risks, motivating the use of machine vision, machine learning, and automated risk assessment (59, 72–74). Beyond manufacturing, these technologies have expanded to construction, logistics, agriculture, healthcare, and pharmaceutical environments (44, 75–79), demonstrating cross-sector applicability. These applications indicate that human-centered Industry 5.0 technologies can be adapted to diverse work settings where physical workload, repetitive tasks, human–technology interaction, and worker safety represent critical occupational concerns.

In this sense, the evolution observed across the studies suggests a transition from technology-assisted ergonomic assessment toward intelligent, predictive, and adaptive approaches to occupational risk management. Early applications primarily demonstrated the feasibility of individual technologies for assessing posture, monitoring physical workload, or supporting safer task execution, whereas more recent studies increasingly combine multiple sensing modalities with artificial intelligence, collaborative robotics, digital twins, and adaptive decision-making (52, 53, 60, 80–82). This convergence enables continuous monitoring of worker conditions and supports earlier identification of changes in workload, fatigue, posture, and other occupational risk factors (55, 57). Accordingly, the emerging technological paradigm extends beyond the automation of ergonomic assessment toward work environments capable of detecting worker conditions, interpreting individual needs, predicting potential risks, and supporting timely preventive or adaptive modifications to tasks and system configurations.

### 3.4 Human-centered industry 5.0 technology used in ergonomic and occupational risks

A diverse set of Human-Centered Industry 5.0 technologies was identified for the assessment and reduction of ergonomic and occupational risks across the included industrial environments. Artificial intelligence was applied to risk prediction, fatigue detection, stress assessment, and adaptive decision-making (53, 72, 80, 83, 84). Computer vision was used for automated posture recognition, movement analysis, and ergonomic risk classification (59, 62, 85, 86). Wearable technologies, including IMUs, smartwatches, smart garments, and electromyography, provided biomechanical and physiological information associated with worker exposure (52, 54–56, 87). Digital twins supported workload simulation, fatigue assessment, and task allocation (39, 88–90). Exoskeletons and collaborative robots were primarily applied to physical workload reduction and task redistribution (42, 44, 45, 48, 91). Extended reality and IIoT systems supported ergonomic assessment, worker training, connected monitoring, and workstation design (70, 92, 93).

The reported applications covered multiple occupational risk dimensions, including hazardous postures, musculoskeletal exposure, physical workload, fatigue, physiological stress, cognitive workload, and human–robot interaction. Postural and movement risks were predominantly assessed using computer vision, wearable sensors, and artificial intelligence (40, 59, 62, 85, 86). Physical workload and fatigue were addressed through exoskeletons, digital twins, physiological monitoring, and adaptive task allocation (44, 48, 88, 89, 94). Cognitive and physiological conditions were increasingly evaluated through multimodal sensing involving wearable devices, eye tracking, artificial intelligence, and human–robot interaction systems (51, 54, 56, 60). Several studies also combined complementary technologies, including exoskeletons with sEMG, cobots with inertial and physiological sensors, computer vision with wearable devices, and AI with digital twins (42, 55, 57, 69, 90, 93–95). These integrated configurations enabled simultaneous assessment of multiple worker-related conditions and production variables (Figure 4).

**Figure 4:**
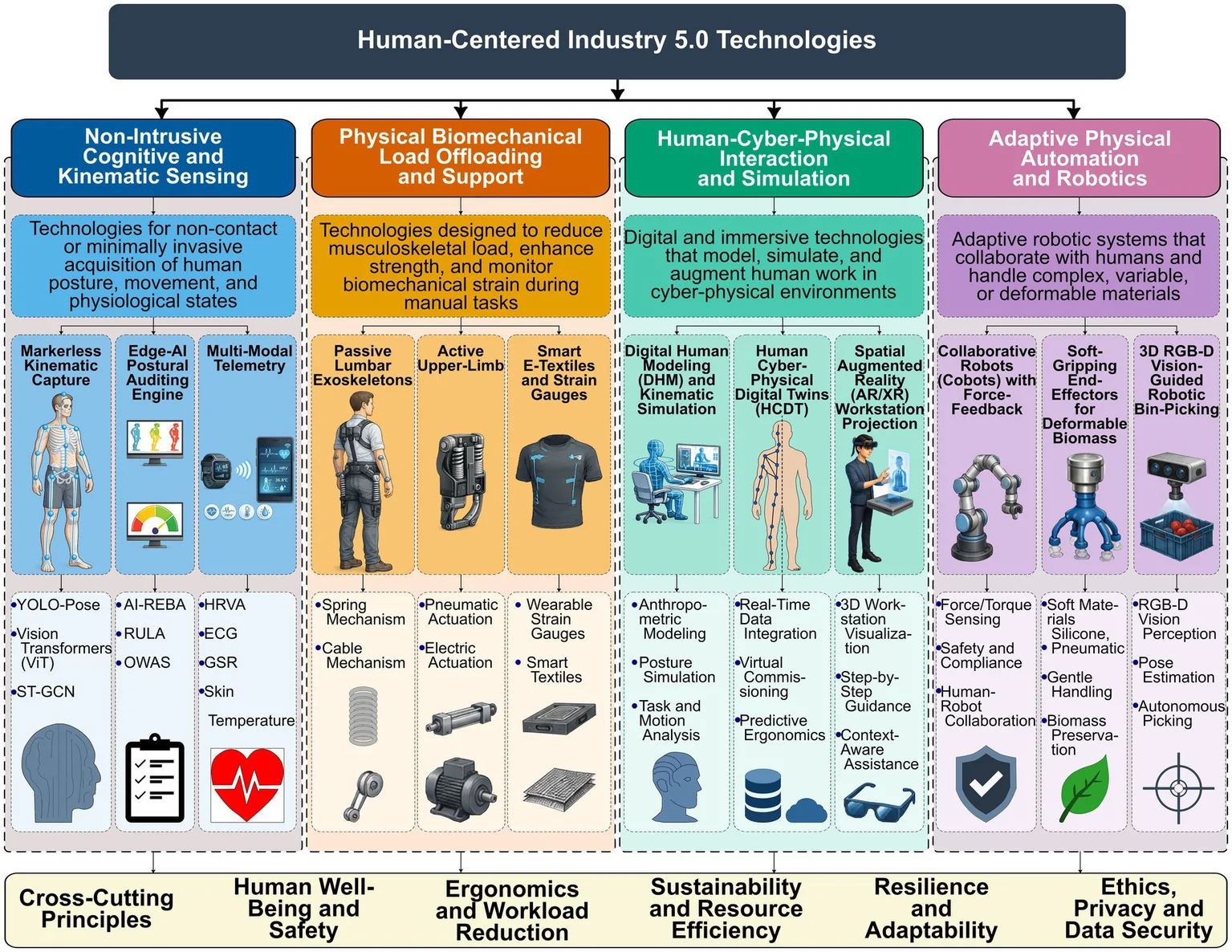
Taxonomy of human-centered Industry 5.0 technologies synthesized from the systematic literature review ( N=78studies ). The framework organizes interventions into four core functional domains: (1) Non-Intrusive Cognitive and Kinematic Sensing, comprising markerless kinematic capture, edge-AI postural auditing engines, and multi-modal telemetry; (2) Physical Biomechanical Load Offloading and Support, spanning passive lumbar exoskeletons, active upper-limb devices, and smart e-textiles and strain gauges; (3) Human-Cyber-Physical Interaction and Simulation, encompassing Digital Human Modeling (DHM) and kinematic simulation, Human Cyber-Physical Digital Twins (HCDT), and spatial Augmented Reality (AR/XR) workstation projection; and (4) Adaptive Physical Automation and Robotics, covering collaborative robots (cobots) with force-feedback, soft-gripping end-effectors for deformable biomass, and 3D RGB-D vision-guided robotic bin-picking. The bottom banner outlines the cross-cutting Industry 5.0 principles unifying these technological branches: human well-being and safety, ergonomics and workload reduction, sustainability and resource efficiency, resilience and adaptability, and ethics, privacy, and data security.

### 3.5 Contributions to worker health, safety and operational performance

Postural assessment was one of the most extensively investigated applications, using computer vision, wearable sensors, digital twins, and skeleton-based recognition to identify postures associated with occupational ergonomic risk. Human-centered digital twins achieved 79.63% Top-1 accuracy compared with 21.78% for conventional 2D recognition under limited worker visibility (39). Smartwatch–LiDAR fusion achieved approximately 98% mean posture-recognition accuracy for upper- and lower-body assessment (85), while monocular RGB systems reached 81.90% mean angular accuracy for static postures (62). More advanced architectures further improved automated assessment, with PRoTAS achieving 94.52% F1-score and AUC of 1.000 (40), and Vision Transformers reporting AUC > 0.996 for real-time posture classification (86). These findings support increasingly accurate and continuous occupational risk surveillance in Supplementary Table S3.

Industry 5.0 technologies were increasingly used not only to identify biomechanical exposures but also to modify job demands through physical assistance. Upper limb exoskeletons reduced muscle activity by 25.8–51.8% in the deltoid and biceps during repetitive overhead activities, while perceived usability and comfort reached 4.13/5 and 4.63/5, respectively (44). Other passive upper limb exoskeletons reduced anterior and medial deltoid activity by 21.6 and 13.6%, respectively, with 62% of participants achieving System Usability Scale scores above 80 (48). Another study reported reductions in muscle activity of 46.46, 34.11, and 44.72% (94). These findings indicate possible reductions in biomechanical exposure during demanding tasks.

Manual handling and repetitive work were also addressed through human–robot collaboration and intelligent task allocation, enabling physical demands to be redistributed between workers and robotic systems. One study assigned 47% of operations to cobots, reducing energy expenditure and balancing workers’ physical workload through genetic-algorithm-based job rotation (65). Another learning–forgetting and task-allocation model reduced production time by 10–15% compared with the conventional LFFR-HRC approach and by 25–35% compared with manual systems, while incorporating energy expenditure and rest allowances into task allocation (96). Collaborative workstation optimization based on Pareto analysis simultaneously considered task allocation, REBA risk, and fatigue, obtaining cycle times of 22.5–25.1 min/unit, REBA = 6.56, and estimated fatigue of 60.7% (57). These findings indicate that occupational risk prevention is increasingly integrated into production planning.

Fatigue monitoring constituted another prominent application, particularly through Human Digital Twins, physiological sensing, computer vision, and machine learning. Fatigue-aware task planning using a Human Digital Twin reduced maximum fatigue from 0.89 to 0.38 and mean fatigue from 0.22 to 0.10, while the Modified Borg score decreased from 4.4 to 2.2 and fatigue onset was delayed from 4.5 to 6.3 rounds (88). A multimodal system combining a Human Digital Twin, computer vision, wearable sensors, and genetic optimization achieved 99.97% frame accuracy and 100% target-arm identification; the optimized scheduling strategy reduced maximum fatigue to 45.5% while maintaining a 1614.6 s cycle time (89). These findings indicate that integrated technologies can support continuous fatigue monitoring and enable adaptive task planning to reduce worker exposure.

Similarly, computer vision combined with expert systems demonstrated real-time fatigue estimation, with the ECAConv–LSTM architecture achieving MSE = 0.028, R^2^ = 0.941, and an inference time of 0.71 ms/frame (72). Digital-twin-based monitoring using EWMA control charts detected fatigue in 78.5% of left-back, 71% of left-elbow, and 64% of right-knee experimental conditions (97). Physiological approaches complemented these systems, with fNIRS-based monitoring demonstrating strong relationships between physical fatigue and cerebral oxygenation, while metabolic energy expenditure also showed associations with cerebral oxygenation (43). Collectively, these findings indicate a transition toward predictive fatigue management, in which continuous physiological and ergonomic information can support earlier identification of fatigue and inform adaptive task scheduling, work allocation, and preventive interventions aimed at reducing excessive worker exposure.

Multimodal monitoring further expanded occupational risk assessment from physical exposure toward combined physical and cognitive dimensions. During packaging operations, pulse rate was associated with perceived exertion, electrodermal activity increased across shifts, and the visual-fatigue component explained 93.64% of the observed variance (75). Wearable ECG monitoring combined with a one-dimensional convolutional neural network achieved 88.4% accuracy and an *F1*-score of 0.90 for real-time stress detection (56), whereas wearable physiological signals combined with XGBoost achieved 99.70% accuracy and an MCC of 0.994 for worker-stress prediction (61). These findings demonstrate that Industry 5.0 technologies can extend occupational risk assessment beyond observable physical conditions by continuously monitoring physiological responses associated with fatigue, stress, and cognitive workload, thereby supporting earlier identification of potential risks and more timely preventive interventions during work activities.

Cognitive workload and human factors were particularly relevant in collaborative environments where workers interact directly with robots or automated systems. Eye-tracking and physiological measurements were used to characterize visual attention, workload, and stress during human–robot interaction. In one study, dual-task conditions increased errors from 0.81 to 5.64 and blink rate from 14.87 to 23.30 min^−1^, with heart rate reaching approximately 101 bpm; the predictive model explained 61% of the variance (51). Another eye-tracking study reported a significant reduction in NASA-TLX workload, while worker acceptance was associated with perceived stress (64). Multimodal biosensing during collaborative robot programming identified EEG theta activity (76.67%), alpha activity (70.53%), GSR recovery (72.86%), and HRV PNN25 (71.58%) as stress-related indicators (54). EEG–fNIRS with machine learning achieved adjusted R^2^ = 0.654 and 77.8% classification accuracy for cognitive workload prediction (60).

Human–robot interaction was also investigated from the perspectives of usability, worker adaptation, and workload management. Personalized HRC workload management reduced average task time from 11.91 to 9.46 s while monitoring changes in heart rate from 78.49 to 82.10 bpm, indicating that adaptive collaboration can respond simultaneously to worker characteristics and operational requirements (98). A Human–Robot Multistation System integrating gesture-based guidance and adaptive scheduling reduced worker travel distance by 70%, while achieving SUS = 70.5 and NASA-TLX = 37 (67). In addition, automated arm-kinematic estimation using IMU sensors and machine learning achieved R^2^ values of 0.9968–0.9973 and RMSE values of 0.6338–2.7721, providing accurate biomechanical information for occupational risk monitoring (52). These findings indicate that human–robot collaboration is increasingly being configured as an adaptive system integrating worker state, movement, workload, and task requirements.

Workplace redesign constituted a further application in which occupational risk information was incorporated directly into production-environment design. Digital ergonomic assessment combined with RFID-enabled manufacturing identified 47 EAWS trunk-risk points, with trunk rotation accounting for 73.1% of task duration (125.5/171.7 s), providing specific evidence for workstation intervention (93). Virtual reality and motion capture were used to evaluate workplace configurations and identify significant differences in head and trunk postures ( p<0.05 ), particularly during extreme flexion and rotation (70). Similarly, surrogate modelling for adaptive human-centered workstations identified 52.8% high-risk postures, with awkward postures contributing 68.9% of EAWS scores (82). A Kansei Engineering and axiomatic-design approach reduced information content from 8.19 to 4.10 bits, supporting safer HRC workstation configurations (99). These findings indicate that occupational risk assessment is increasingly incorporated upstream into production-system design, enabling preventive modifications before risks become embedded in routine operations.

Evidence shows that human-centered Industry 5.0 technologies are being applied across six interconnected ergonomic domains: postural assessment, manual material handling, fatigue monitoring, cognitive workload, human-robot interaction, and work-place redesign (Figure 5). Physical ergonomics remains the dominant dimension, but the increasing integration of physiological and behavioral measurements demonstrates a progressive expansion into cognitive and psychosocial ergonomics. Importantly, the most advanced applications not only detect ergonomic risk but also connect assessment results with task allocation, robotic assistance, workplace adaptation, and predictive decision-making. Consequently, evidence supports a transition from reactive ergonomic control to sustain-able, human-centered systems capable of continuously balancing worker well-being, safety, and production requirements (57, 61, 65, 82, 88, 89, 96–98).

**Figure 5:**
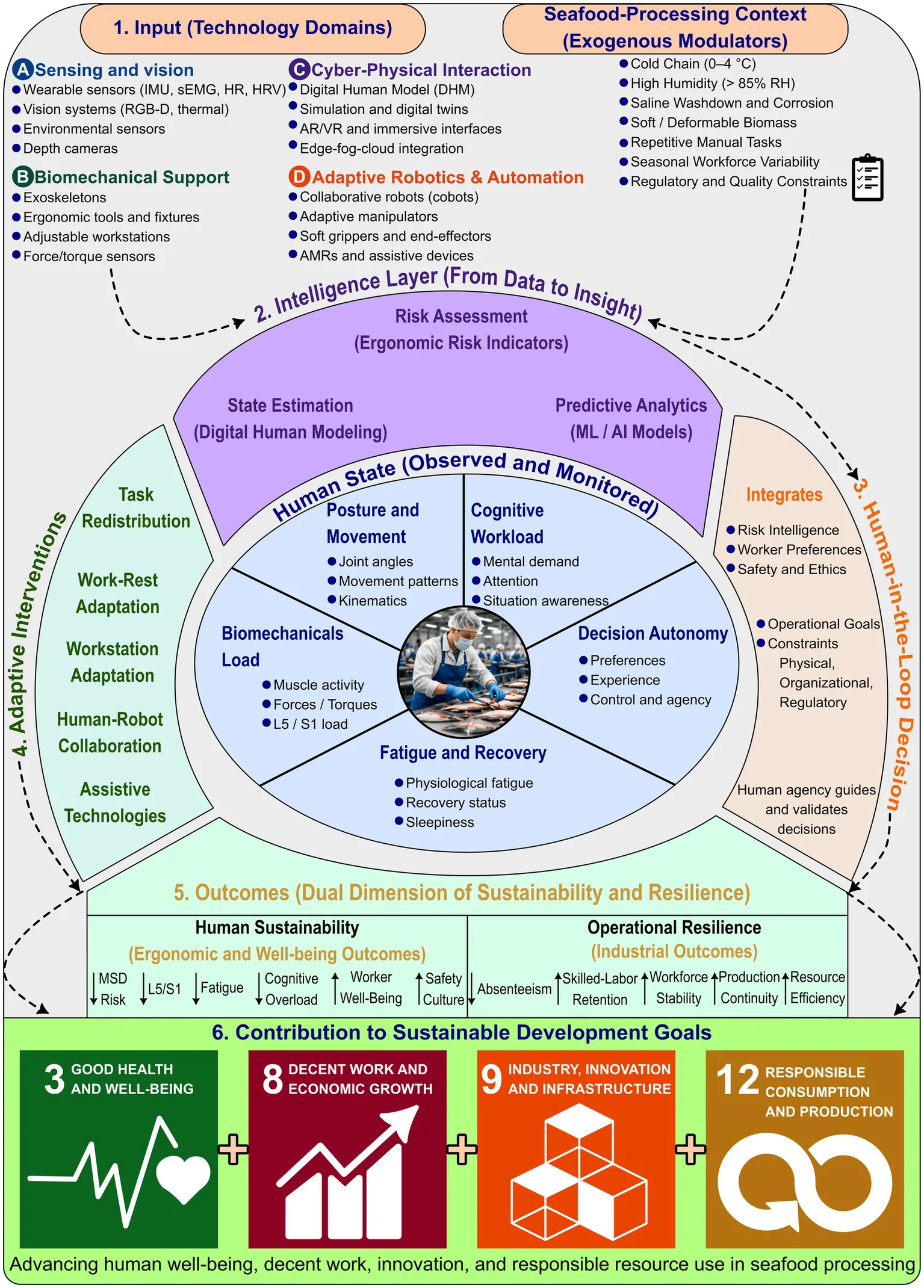
Evidence-derived human-in-the-loop adaptive framework for sustainable ergonomics and operational resilience. The architecture organizes findings into a closed-loop system across six sequential steps: (1) Input (Technology Domains) and the Seafood-Processing Context (Exogenous Modulators), establishing ambient, operational, and physical constraints; (2) Intelligence Layer (From Data to Insight), which processes observed human states (posture, cognitive workload, biomechanics, fatigue) through risk assessment, state estimation, and predictive analytics; (3) Human-in-the-Loop Decision, integrating risk intelligence and worker preferences to guide operational goals; (4) Adaptive Interventions, which trigger targeted actions such as task redistribution, work-rest adaptation, and hu-man-robot collaboration; (5) Outcomes (Dual Dimension of Sustainability and Resilience), balancing ergonomic human sustainability with operational industrial outcomes; and (6) Contribution to Sustainable Development Goals, specifically aligning the framework with SDGs 3, 8, 9, and 12 to advance human-centered Industry 5.0 systems.

## 4 Discussions

### 4.1 From risk detection to human-centered occupational risk reduction

The cross-sectoral evidence indicates a transition from isolated risk detection toward integrated approaches that combine monitoring, prediction, and adaptive intervention. Computer vision, wearables, digital twins, collaborative robots, exoskeletons, and artificial intelligence increasingly support earlier identification of occupational risks and more timely preventive responses (39, 69, 84, 85, 90). For example, IMU-based assessment reduced evaluation time from 70 to 2 min while maintaining comparable RULA classifications (87), while AI-based systems achieved high performance in real-time posture and fatigue recognition (40, 72, 86).

However, technological sophistication alone does not guarantee occupational risk reduction. Evidence from digital twins, physiological sensing, and adaptive task allocation suggests that effectiveness depends on integrating worker conditions with task and system requirements (81, 88, 89), Therefore, the central contribution of Industry 5.0 lies not simply in continuous monitoring, but in linking risk information with appropriate human-centered interventions while maintaining worker safety and operational requirements.

### 4.2 Comparative effectiveness of emerging technologies

The evidence indicates that the effectiveness of Industry 5.0 technologies for occupational risk reduction depends less on technological novelty than on their capacity to transform risk assessment into continuous, objective, and actionable intervention. Computer vision and AI-based systems showed strong performance for postural risk assessment, with recent systems achieving F1 scores above 0.99 and AUC above 0.996 for real-time posture classification (86), while PRoTAS reached an F1 score of 94.52% at 15–20 fps (40). Wearable systems provided complementary advantages by capturing physiological and biomechanical information not reliably inferred from external posture alone. IMU-based assessment reduced evaluation time from 70 to 2 min while maintaining comparable RULA classifications (87), whereas multimodal wearables captured physiological responses associated with exertion and fatigue (75). Thus, computer vision appears particularly suitable for posture and movement monitoring, whereas wearables offer greater value for physiological risk assessment; however, vision remains sensitive to occlusion and environmental conditions, while wearables may affect comfort and worker acceptance.

Digital twins and adaptive AI systems demonstrated a different level of potential for occupational risk reduction because they extend assessment toward prediction and intervention. Human digital twins combined with fatigue models reduced maximum fatigue from 0.89 to 0.38 and delayed fatigue onset from 4.5 to 6.3 rounds (88), while another multimodal digital-twin approach achieved 99.97% frame accuracy and reduced maximum fatigue to 45.5% through optimized human–robot scheduling (89). Similarly, reinforcement-learning approaches incorporated fatigue into task allocation, reducing fatigue violations while maintaining 100% task completion (81). These findings suggest that predictive technologies may offer greater preventive value than systems limited to risk classification because they can modify task allocation before excessive exposure occurs. However, their occupational safety maturity remains constrained by dependence on training data, simulation assumptions, model reliability, and limited validation under real workplace conditions.

In contrast, cobots and exoskeletons provide more direct physical intervention, with exoskeleton studies consistently reporting reductions in muscle activity of approximately 21.6–51.8% (44, 48) and up to 46.46% for specific upper-limb muscles (94), while collaborative robotics reduced cycle time and redistributed demanding tasks (41, 100). However, combined human–robot technologies do not necessarily produce additive occupational safety benefits; one study reported a 7% cycle-time reduction with a cobot alone but a 17% increase when combined with an exoskeleton (42). This finding indicates that effectiveness depends on task–technology fit, worker characteristics, system configuration, and interaction design rather than simply accumulating technologies. XR technologies appear comparatively mature for training, visualization, and workstation design, but their contribution to continuous occupational risk reduction remains less established because benefits depend strongly on task context and require validation under prolonged real-world industrial conditions (50, 70, 101).

### 4.3 Human factors and technology adoption

The cross-sectoral evidence suggests that human-centered digital transformation can strengthen operational resilience by integrating worker conditions into production management. Technologies that continuously monitor fatigue, redistribute tasks, or support adaptive intervention may reduce the likelihood that worker-related constraints disrupt production. Dynamic human–robot allocation-maintained production under energy constraints (45), reinforcement-learning approaches achieved 100% task completion while reducing fatigue violations (81), and remote-controlled cobots maintained 100% task efficiency and quality under workforce constraints (102). Collaborative line balancing reduced cycle time by 23–28.4% (100), while wearable-assisted HRC and AR/VR applications improved efficiency by approximately 6 and 23.61%, respectively (50, 95). These findings indicate that operational resilience is strengthened when worker safety and adaptive risk management are incorporated into production planning rather than treated as separate functions.

An important distinction is therefore between technologies that improve efficiency under stable conditions and those that support adaptation to changing occupational and production conditions. Digital twins, fatigue-aware scheduling, and reinforcement learning may provide greater adaptive potential because they dynamically incorporate worker or process states (81, 84, 90). The latter may provide stronger support for operational resilience because their value extends beyond predefined configurations. However, most evidence remains limited by laboratory experiments, simulations, single-case applications, and short-term evaluations (45, 84, 90, 100). From an occupational health perspective, human-centered digital transformation could strengthen resilience by linking worker-state monitoring with adaptive task allocation, collaborative automation, and timely intervention when fatigue, workload, personnel availability, or process conditions change.

### 4.4 Implications for occupational health and safety

These technological developments also present potential implications for occupational health and safety that should be considered alongside their ergonomic benefits. Advanced artificial intelligence, Vision Transformers, and computationally intensive digital twins may increase system complexity and require continuous monitoring infrastructures (89, 90). Wearable sensors and smart devices can generate additional demands related to comfort, maintenance, and worker acceptance (69, 87). Moreover, continuous physiological and behavioral monitoring may create concerns regarding privacy, perceived surveillance, and worker autonomy, particularly when collected data are used primarily for productivity control (45, 56). Therefore, human-centered Industry 5.0 should not be evaluated solely according to its capacity to reduce ergonomic exposure. Its implementation should also ensure responsible data governance, transparency, worker participation, privacy protection, and safeguards against excessive technological monitoring.

### 4.5 Cross-sectoral transferability: deploying industry 5.0 in unstructured and harsh operational environments

Technology transfer is particularly relevant to unstructured and demanding work environments, where humidity, low temperatures, wet surfaces, frequent cleaning, variable lighting, and personal protective equipment may affect both technological performance and occupational safety. Machine vision systems have demonstrated strong capabilities for postural classification and ergonomic risk assessment (40, 86), while wearable devices and exoskeletons provide complementary physiological and biomechanical information (75, 87). However, occlusion, condensation, camera positioning, battery limitations, and compatibility with hygiene protocols may restrict their direct application in seafood processing. Therefore, transfer requires technologies capable of maintaining reliable risk assessment under harsh conditions while preserving worker comfort, mobility, hygiene, privacy, and safe interaction with equipment during prolonged production activities.

Based on cross-sector evidence, an evidence-informed technology transfer pathway is proposed for seafood processing a less addressed sector with high impact. This pathway encompasses the identification of ergonomic risks, technologies selection, contextual adaptation, and human-centered validation. Technologies that demonstrate effectiveness in conventional manufacturing cannot be assumed to perform similarly in seafood processing, as environmental, hygienic, operational, and labor conditions may alter their performance. Therefore, the proposed roadmap (Figure 6) comprises four stages: identification of critical tasks and occupational hazards; selection of technologies based on their demonstrated capabilities; adaptation to environmental and operational limitations; and validation under real-world production conditions using integrated indicators of ergonomic exposure, worker safety, usability, and operational performance. This roadmap should be considered a structured proposal for future validation rather than an experimentally demonstrated transfer framework for seafood processing.

**Figure 6:**
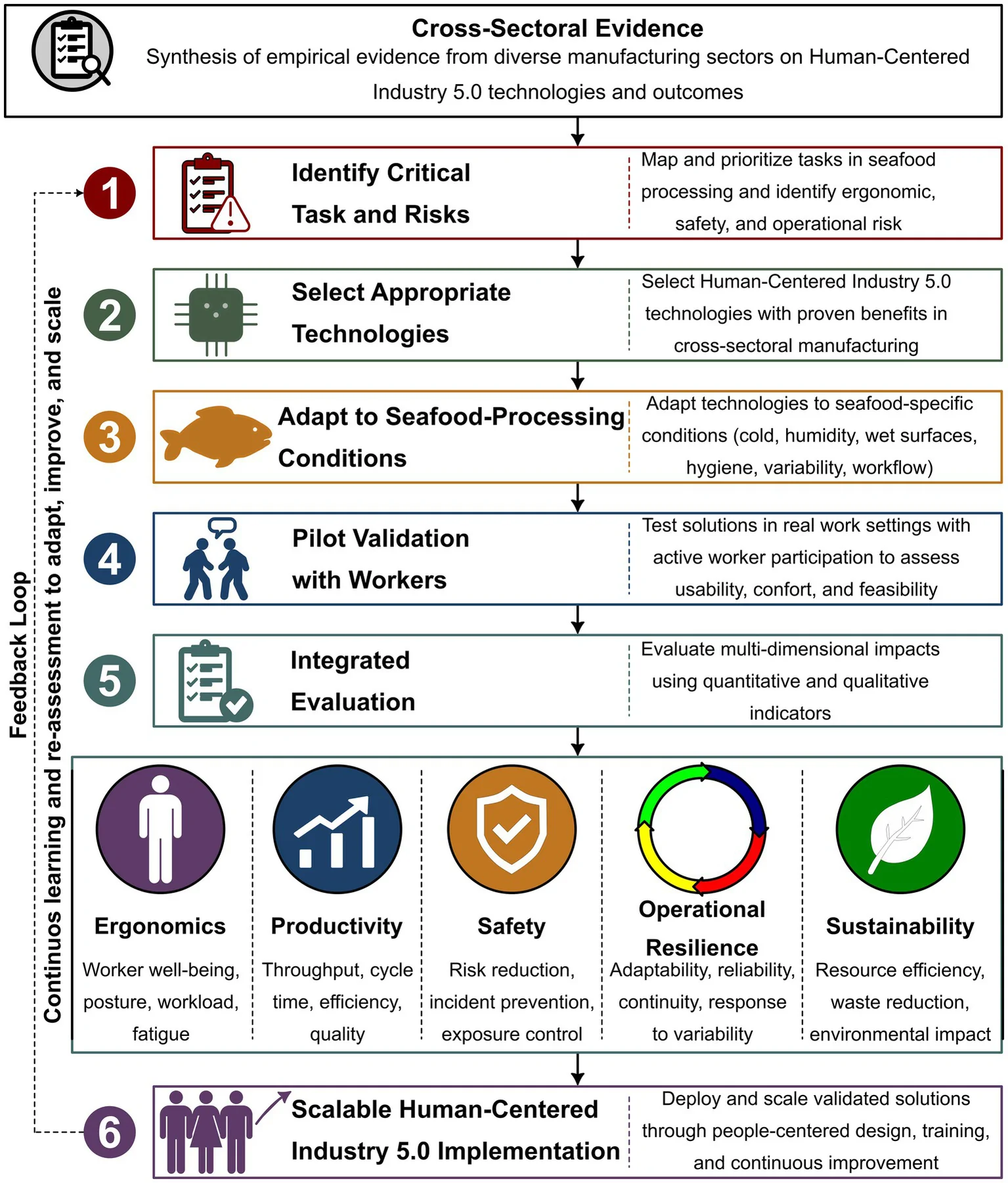
Structured roadmap for the cross-sectoral transfer and scalability of human-centered Industry 5.0 evidence to the seafood-processing industry. The methodology follows a six-step pipeline: (1) Identify Critical Tasks and Risks, mapping ergonomic, safety, and operational hazards under specific processing constraints; (2) Select Appropriate Technologies, leveraging technologies with demonstrated capabilities in other industrial sectors; (3) Adapt to Sea-food-Processing Conditions, adjusting technologies to withstand harsh environments (cold, humidity, wet surfaces, hygiene, and high workflow variability); (4) Pilot Validation with Workers, conducting in-situ usability, comfort, and feasibility testing; (5) Integrated Evaluation, measuring multidimensional impacts across five core dimensions: Ergonomics, Productivity, Safety, Operational Resilience, and Sustainability; and (6) Scalable Hu-man-Centered Industry 5.0 Implementation, following successful validation through people-centered design and training. The continuous feedback loop ensures ongoing learning, reassessment, adaptation, and scaling.

### 4.6 Research gaps, practical implications, and future directions

Future research should prioritize standardized and interoperable frameworks that integrate ergonomic, technological, and occupational safety indicators within Industry 5.0 systems. Current heterogeneity in assessment protocols, sensor configurations, artificial intelligence metrics, and outcome criteria limits direct comparison and transferability across industrial environments (49, 83, 93). Researchers should therefore evaluate technologies not only according to technical performance but also worker acceptance, usability, safety, interoperability, and real-world applicability (50, 64, 95, 100). Worker participation should be incorporated into technology selection and validation to support human-centered implementation (64, 103).

Future research should also move from isolated technological demonstrations toward integrated and predictive systems for occupational risk prevention. Human Digital Twins could combine biomechanical, physiological, cognitive, and contextual information to support dynamic task allocation and fatigue prediction (88–90). Explainable AI, Edge AI, and multimodal models may further improve interpretability, low-latency monitoring, and adaptive work guidance (42, 75). However, these approaches require longitudinal and real-world validation before their effectiveness can be generalized across occupational environments.

## 5 Conclusion

This systematic review demonstrates that Human-Centered Industry 5.0 technologies can support occupational risk reduction by integrating continuous assessment, predictive analytics, physical assistance, adaptive task allocation, and human–technology collaboration. Artificial intelligence, computer vision, wearable sensors, digital twins, collaborative robots, exoskeletons, and extended reality showed measurable capabilities for identifying ergonomic risks, reducing physical workload and fatigue, assessing cognitive demands, and supporting safer task and workstation design. Their greatest potential emerges when these technologies are integrated into human-centered sociotechnical systems rather than deployed as isolated solutions. Nevertheless, methodological heterogeneity, laboratory-based validation, short-term evaluations, and limited longitudinal assessment constrain conclusions regarding sustained occupational health and safety benefits. Cross-sector findings provide a basis for transferring these solutions to demanding environments such as seafood processing, although environmental, operational, hygienic, and workforce conditions require context-specific validation. Future research should prioritize participatory design, predictive risk management, interoperable technologies, worker privacy, and real-world validation to ensure that Industry 5.0 delivers measurable improvements in worker health, safety, and sustainable work systems.

## Data Availability

The original contributions presented in the study are included in the article/Supplementary material, further inquiries can be directed to the corresponding author.

## Footnotes

1
https://estech.shinyapps.io/prisma_flowdiagram/
