# Supplementary material for: Human-centered industry 5.0 technologies for occupational risk reduction: a systematic review
Source: Front Public Health. 2026 Oct 1;14:1976362. doi: 10.3389/fpubh.2026.1976362 (PMC13668588; doi:10.3389/fpubh.2026.1976362)
Supplement: Supplementary file 1 [file Data_Sheet_1.zip › Table S3. Applications of Human-Centered Industry 5.0 Technologies for Sustainable Ergonomics..docx]

**Table S3.** Applications of Human-Centered Industry 5.0 Technologies for Sustainable Ergonomics.

| **Technology** | **Ergonomic application** | **Ergonomic dimension** | **Assessment method** | **Main outcomes** | **Ref.** |
| --- | --- | --- | --- | --- | --- |
| Human-Centered Digital Twin with Computer Vision | Human motion recognition and ergonomic monitoring in collaborative workcells | Physical ergonomics | Cross-view skeleton-based action recognition (STGCN-2D/STGCN-3D) | STGCN-3D achieved 79.63% Top-1 accuracy versus 21.78% for STGCN-2D under 50% observation; metrics averaged over five independent runs. | (39) |
| Collaborative Robotics (Cobots) | Human–robot collaborative assembly workstation design | Physical ergonomics | Digital simulation, RULA and NIOSH Lifting Index (LI) | Cycle time decreased from 7 min 39 s to 6 min 55 s (9.6% reduction); critical human tasks reduced from 5 to 2; maximum RULA (4) and LI (2) remained unchanged. | (40) |
| Human Digital Twin (HDT) | Fatigue-aware task planning in human–robot collaborative assembly | Physical ergonomics | Fatigue model, Modified Borg Scale and Wilcoxon signed-rank test | Optimization reduced maximum fatigue (0.89–0.38), mean fatigue (0.22–0.10), Borg score (4.4–2.2), and delayed fatigue onset (6.3 vs. 4.5 rounds; p = 0.0049). | (41) |
| Wearables (Smartwatch) + LiDAR Sensor Fusion | Real-time postural or proximity monitoring | Physical ergonomics | Smartwatch inertial sensors, LiDAR and ISO 11226 | Achieved ≈98% mean posture recognition accuracy for upper- and lower-body assessment, enabling privacy-preserving detection of WMSD risk. | (42) |
| Exoskeletons and Collaborative Robots (Cobots) | Physical assistance and collaborative assembly | Physical ergonomics | RULA assessment, cycle time analysis and sentiment analysis | RULA scores improved by 11% (up to 28% upper-limb reduction); cycle time decreased to 78 s (−7%) with cobot but increased to 98 s (+17%) with combined technologies. | (44) |
| Multimodal Wearable Sensors and Eye Tracking | Multidimensional ergonomic assessment during packaging operations | Physical and cognitive ergonomics | Borg Scale, pulse rate, EDA, RTLX, Tobii Pro Glasses 3 and PCA | Pulse rate correlated with perceived exertion (ρ=0.266, p<0.001); Electrodermal activity (EDA) increased across shifts (ρ=0.228, p<0.001); visual fatigue (PC2) explained 93.64% variance (ρ=0.529, p<0.001). | (45) |
| Computer Vision (Monocular RGB Camera) | Automated posture monitoring and ergonomic risk assessment | Physical ergonomics | Monocular RGB imaging, deep learning and RULA | Mean angular accuracy reached 81.90% (static) and 75.68% (frontal dynamic); RULA Grand Score and Risk Level accuracies achieved 74.73% and 77.87%, respectively. | (46) |
| Kansei Engineering + Axiomatic Design Systems | Product/workstation design based on emotional/perceptual needs | Cognitive / Psychological | Kansei evaluation and axiomatic mapping | Applying Suh's Axiom 2 reduced Information Content from 8.19 to 4.10 bits, supporting independent ergonomic design parameters for physically and cognitively safe HRC workstations. | (47) |
| Wearable Sensors (IMU) | Automated assessment of work-related musculoskeletal disorder (WMSD) risk | Physical ergonomics | Inertial Measurement Units (IMUs) and RULA comparison | Reduced assessment time from 70 to 2 min while achieving comparable RULA classification (Level 2: 88% vs. 86%; Level 3: 1% vs. 3%; Level 4: 0%). | (48) |
| Neuroergonomics (fNIRS) | Physical fatigue assessment during manual automotive assembly | Physical ergonomics | fNIRS cerebral oxygenation, NIOSH, OWAS, RULA, ANOVA | Physical fatigue strongly correlated with cerebral oxygenation (r = −0.938, p < 0.001); metabolic energy expenditure also correlated with cerebral oxygenation (r = −0.924, p < 0.001). | (50) |
| Upper Limb Exoskeletons | Upper-limb assistance during repetitive overhead tasks | Physical ergonomics | Surface electromyography (%MVC) and comfort | Reduced muscle activity by 25.8–51.8% (%MVC) across deltoid and biceps muscles (p = 0.002–0.006); perceived usability reached 4.13/5 and comfort 4.63/5. | (51) |
| Computer Vision + AI Datasets | Human behavior understanding and ergonomic posture monitoring | Physical ergonomics | RGB-D vision, deep learning, EAWS, OpenPose and MocapNet2 | PRoTAS achieved 94.52% recall, 94.52% F1-score and AUC = 1.000; the framework operated in real time at 15–20 fps with 50–67 ms latency. | (52) |
| Human Digital Twin, Computer Vision and Wearable Sensors | Fatigue-sensitive task planning in human–robot collaborative assembly | Physical and cognitive ergonomics | MS-TCN, LSTM, Fitbit Sense 2 and genetic algorithm | Achieved 99.97% frame accuracy and 100% target-arm identification; optimized HRC scheduling reduced maximum fatigue to 45.5% with a 1614.6 s cycle time. | (53) |
| Cobots + Eye Tracking | Visual attention and cognitive workload tracking during robotic interaction | Cognitive ergonomics | Eye-tracking (fixation/blink metrics), heart rate, NASA-TLX, regression analysis | Dual-task increased errors (5.64 vs 0.81), blink rate (23.30 vs 14.87 min⁻¹), HR ≈101 bpm; prediction model R²=0.61. | (55) |
| AI + Wearable Sensors (IMU / Electrodes) | Estimation of upper limb kinematics for ergonomic monitoring | Physical ergonomics | IMU-based motion capture, Random Forest regression, R² and RMSE validation | Predicted arm motion with R²=0.9968–0.9973 and RMSE=0.6338–2.7721, demonstrating highly accurate biomechanical estimation and robust generalization. | (57) |
| Computer Vision + Expert Systems | Real-time fatigue assessment from workers' posture in smart manufacturing environments | Physical ergonomics | MediaPipe pose estimation, deep learning (CNN, LSTM, CNN–LSTM, ECAConv–LSTM), MSE, R², inference time | Hybrid ECAConv–LSTM achieved MSE=0.028, R²=0.941, and 0.71 ms/frame inference, enabling accurate real-time fatigue assessment. | (60) |
| Machine learning + surface EMG sensors | Recognition of arm muscle activation during manual assembly tasks | Physical ergonomics | Two-channel EMG, machine learning classification | Classified four assembly movements with 89.5% accuracy using 2-channel EMG, enabling reliable muscle activity recognition while reducing sensor requirements. | (62) |
| Kinect skeleton tracking + machine learning | Automatic assessment of worker performance and cycle-time monitoring during assembly operations | Physical ergonomics | Kinect skeletal tracking, kNN clustering, linear perceptron, motion pattern mining | Detected work cycles of 25.8–44.5 s (200–345 frames); motion thresholds 0.2–0.8 m/s enabled automated ergonomic performance assessment. | (63) |
| Cobots + Multimodal Bio-sensors (HRV, GSR, EEG) | Assessment of operator stress during collaborative robot programming | Cognitive ergonomics | EEG, ECG, GSR, facial expression analysis, NASA-TLX correlation | EEG Theta=76.67%, Alpha=70.53%, GSR recovery 72.86%, HRV PNN25=71.58%, showing strongest agreement with perceived stress. | (64) |
| Eye-tracking + collaborative robot workstation assessment | Evaluation of cognitive workload, user acceptance, and trust during human–robot collaboration | Cognitive ergonomics | NASA-TLX scale / Dual-task paradigm metrics | NASA-TLX decreased significantly (χ²=8.24, p<0.05; Z=-2.646, p=0.008); acceptance predicted perceived stress (R²=72.5%, p<0.001). | (65) |
| Passive Upper-Limb Exoskeletons | Reduction of shoulder muscle workload during repetitive overhead industrial tasks | Physical ergonomics | Surface EMG (RMS), QUEST, System Usability Scale (SUS) | Reduced anterior and medial deltoid EMG (−21.6%, −13.6%); 62% achieved SUS>80 and 100% reported positive QUEST satisfaction. | (69) |
| Electro-pneumatic arm exoskeleton with EMG control | Passive upper-limb rehabilitation through biomechanical assistance and motion control | Physical ergonomics | EMG calibration, angular displacement validation, simulation–prototype comparison | Achieved 74.5° motion with 11.96% overall error; flexion errors 2.08–11.30%, extension errors 2.00–16.67%, pneumatic force 18 N. | (70) |
| Wearable body temperature sensors (Cosinuss, VivaLNK, Equivital) | continuous monitoring of workers' body temperature for health and safety risk detection | Physical ergonomics | Bland–Altman analysis, hospital-grade ear thermometer comparison, comfort survey | VivaLNK showed lowest bias (0.40°C) and agreement range (2.86°C); 73% preferred Equivital for ≥8 h continuous wear. | (72) |
| Data-driven Analytics + Strain Gauges / Vision | Risk assessment for manual tasks involving high repetition in hands/wrists | Physical (Local Biomechanical) | RULA, HAL, BACH, GRU | RULA and HAL classification exceeded 95%; dominant wrist torque significantly higher (p=7.7×10⁻¹³²). | (73) |
| Deep learning + 3D human pose estimation | Recognition of unsafe acts during industrial tasks | Physical ergonomics | Deep learning using 3D human body postures | Achieved 84.7%, 92.0%, and 98.0% accuracy across three manufacturing tasks. | (74) |
| Computer vision + smartwatch sensors + deep learning | Real-time physical strain and fatigue detection | Physical ergonomics | Ergonomic posture classification, heart-rate monitoring, multimodal data fusion | Achieved >70% F1-score; heart rate increased 100–120 s after elevated physical strain. | (75) |
| Modular smart garments + 3D optimization platform | Wearable ergonomic monitoring and sensor integration | Physical ergonomics | 3D optimization, statistical analysis | Outperformed manual placement (p = 0.009 small; p = 0.007 large modules), enabling stable real-time monitoring. | (76) |
| Digital ergonomic assessment + RFID-enabled digital manufacturing | Musculoskeletal risk assessment and workstation redesign | Physical ergonomics | EAWS with RFID-enabled Operator Process Identification (OPI) | Identified 47 EAWS trunk-risk points; trunk rotation occupied 73.1% (125.5/171.7 s) of task duration. | (77) |
| Digital twin + digital human + human–robot collaboration | Dynamic task scheduling and worker assistance | Physical ergonomics | Digital twin validation, t-test, dynamic scheduling | Predicted task time significantly (p < 0.01); worker delay remained <130 s/cycle after dynamic scheduling. | (78) |
| Wearable ECG sensors + 1D CNN + real-time monitoring platform | Real-time stress and productivity monitoring | Cognitive ergonomics | ECG analysis, 1D convolutional neural network (CNN) | Detected stress with 88.4% accuracy and F1-score = 0.90 during real-time monitoring. | (80) |
| Virtual Reality (VR) + Motion Capture (MoCap) | Ergonomic workplace design and posture assessment | Physical ergonomics | Motion capture, RULA comparison | Significant differences (p < 0.05) in head/trunk postures; VR improved detection of extreme flexion and rotation. | (82) |
| Collaborative Workstations (Cobot Integration) | Ergonomic workstation configuration and task allocation | Physical ergonomics | Pareto optimization, REBA, fatigue analysis | Optimal design achieved 22.5–25.1 min/unit, REBA 6.56, and fatigue 60.7%. | (83) |
| Passive lumbar exoskeleton + wearable sEMG + deep learning | Physical fatigue monitoring during manual material handling | Physical ergonomics | Surface EMG, feedforward neural network (FNN), AUC analysis | Achieved 99% classification accuracy (AUC = 1.00); lumbar muscle activation decreased significantly with exoskeleton. | (84) |
| Passive back-support exoskeleton | Physical strain reduction during manufacturing tasks | Physical ergonomics | Perceived Physical Exertion (PPE) | Reduced perceived exertion significantly (F = 8.07, p = 0.0062); mean PPE decreased by 7.25 points. | (85) |
| Eye-Tracking | Visual attention monitoring during repetitive human–robot assembly | Cognitive ergonomics | Eye-tracking (fixation analysis) | Fixations decreased with learning; morning vs afternoon differed significantly (p < 0.001), indicating fatigue effects. | (86) |
| Graph neural networks (G-EAD) with skeleton-based action recognition | Recognition and segmentation of industrial actions to identify repetitive | Physical ergonomics | Graph-based deep learning using 21- and 72-joint skeletal models | G-EAD achieved 73.98% accuracy (21 joints); graph attention improved 74.42% (72 joints). | (88) |
| Wearable robotic exoskeleton | Evaluation of cognitive state, discomfort, and productivity during masonry tasks | Cognitive workload and physical discomfort | Cognitive metrics, productivity, and physical discomfort. | Active exoskeleton improved excitement (68.54%), relaxation (58.40%), and focus (53.10%) while reducing stress (55.74%) | (90) |
| Collaborative robots (cobots) with learning–forgetting and task allocation model (LFFR-HRC + TA) | Intelligent task allocation to reduce physical workload and optimize human–robot collaboration | Physical and organizational ergonomics | Learning–forgetting production model with energy expenditure and rest allowance analysis | Reduced production time by 10–15% versus LFFR-HRC and 25–35% versus manual systems | (91) |
| Computer vision with convolutional neural network (CNN) | Automated upper-limb motion recognition for ergonomic risk prevention movements and gestures | Physical ergonomics | CNN-based pose estimation with kinematic feature extraction and KNN, SVM, and SNN classification | Achieved ≈100% classification accuracy and 1 μs inference, enabling rapid ergonomic risk detection | (92) |
| Human–robot collaboration with genetic algorithm-based job rotation | Optimized task allocation and worker rotation to balance physical workload | Physical / Organizational | Genetic algorithm using energy expenditure and workload distribution analysis | Assigned 47% of operations to cobots, reducing energy expenditure and balancing workers’ physical workload | (93) |
| Vision Transformer (ViT)-based computer vision | Real-time multi-label posture classification for ergonomic risk monitoring | Physical ergonomics | RGB image analysis with ViT and RULA-derived ergonomic labels | Achieved F1 >0.99 and AUC >0.996, enabling real-time ergonomic posture classification | (94) |
| Machine learning with OpenPose and WEKA | Automated working posture assessment and WMSD risk prediction | Physical ergonomics | OpenPose-based computer vision, REBA assessment, and decision tree analysis | Analyzed 11,211 frames; identified 87% medium-risk postures and 365 high-risk cases overlooked conventionally | (95) |
| Digital twin with EWMA control charts | Biomechanical fatigue detection during manufacturing tasks | Physical ergonomics | Digital twin, motion capture, and EWMA statistical control charts | Detected fatigue in 78.5% (left back), 71% (left elbow), and 64% (right knee) experiments | (96) |
| EEG–fNIRS multimodal monitoring with machine learning | Cognitive workload assessment during human–robot collaboration | Cognitive ergonomics | EEG, fNIRS, subjective questionnaires, behavioral measures, regression, ANN, and KNN | Achieved adjusted R² = 0.654 and KNN accuracy of 77.8% for cognitive workload prediction | (97) |
| Motion capture with time-series machine learning | Proactive prediction of upper-limb movements and WMSD risk | Physical ergonomics | Motion capture, SVM-based time-series forecasting, and TMERT risk assessment | Achieved F1 >98% (0.1 s) and >67% (1 s) for proactive WMSD risk prediction | (100) |
| Wearable sensors with XGBoost machine learning | Real-time prediction of worker stress in smart assembly lines | Cognitive ergonomics | Physiological signal monitoring, wearable sensors, and XGBoost classification | Achieved 99.70% accuracy and MCC = 0.994 for real-time worker stress prediction | (101) |
| Upper-limb industrial exoskeleton | Upper-limb muscle load reduction during static and dynamic tasks | Physical ergonomics | Surface electromyography (EMG) and perceived fatigue assessment | Reduced muscle activity by 46.46% (DM), 34.11% (DA), and 44.72% (TD) (p < 0.001) | (102) |
| Surrogate modelling for adaptive human-centered workstations | Continuous ergonomic assessment and adaptive workstation configuration | Physical ergonomics | EAWS-based ergonomic simulation and surrogate modelling | Identified 52.8% high-risk postures; awkward postures contributed 68.9% of EAWS scores | (106) |
| Human–Robot Multistation System (HRMS) | Gesture-based task guidance and adaptive human–robot task scheduling | Cognitive and organizational ergonomics | SUS, Godspeed Questionnaire, NASA-TLX, task completion time, error analysis, and simulation | Reduced travel distance by 70%; SUS 70.5, NASA-TLX 37, with acceptable collaborative usability | (108) |
| Computer vision-based ergonomic risk assessment system | Automated ergonomic risk evaluation in real manufacturing environments | Physical ergonomics | Computer vision with REBA and RULA ergonomic assessment | Achieved 80.0% ergonomic risk classification accuracy using REBA in real manufacturing environments | (110) |
| Virtual reality locomotion system | Evaluation of locomotion techniques during virtual industrial tasks | Cognitive ergonomics | Task performance analysis (response time and errors) | Directed steering reduced response time (0.77–0.73 s) and errors (4.63–3.48; *p* = 0.013) | (111) |
| Personalized human–robot collaboration workload management | Personalized workload adaptation to improve efficiency and worker well-being | Cognitive and physical ergonomics | Heart rate, HRV (RMSSD, HF power), and average task time (AT) | Task time decreased from 11.91 to 9.46 s; heart rate increased from 78.49 to 82.10 bpm | (114) |
| YOLO-HRNet with attention mechanism | Automated real-time ergonomic risk assessment in garment manufacturing | Physical ergonomics | Computer vision with YOLO-HRNet and RULA-based posture assessment | Improved mAP by 4.43% and recall by 5.99%, achieving expert-consistent RULA risk assessment | (116) |

Note. The table shows the applications of human-centered Industry 5.0 technologies for sustainable ergonomics, summarizing the main ergonomic applications, dimensions, evaluation methods, and quantitative results reported in the included studies from 2021 to 2026. Abbreviations: AI, artificial intelligence; AUC, area under the curve; BACH, Baseline Assessment of Cumulative Trauma; CNN, convolutional neural network; ECG, electrocardiography; EDA, electrodermal activity; EEG, electroencephalography; EAWS, European Assembly Worksheet; EMG, electromyography; fNIRS, functional near-infrared spectroscopy; FNN, feedforward neural network; GSR, galvanic skin response; HDT, human digital twin; HR, heart rate; HRV, heart rate variability; HRC, human–robot collaboration; IMU, inertial measurement unit; LSTM, long short-term memory; NASA-TLX, NASA Task Load Index; NIOSH, National Institute for Occupational Safety and Health; OWAS, Ovako Working Posture Assessment System; PCA, principal component analysis; REBA, Rapid Entire Body Assessment; RFID, radio-frequency identification; RULA, Rapid Upper Limb Assessment; SUS, System Usability Scale; WMSD, work-related musculoskeletal disorder; ViT, Vision Transformer; VR, virtual reality.
