# Supplementary material for: Human-centered industry 5.0 technologies for occupational risk reduction: a systematic review
Source: Front Public Health. 2026 Oct 1;14:1976362. doi: 10.3389/fpubh.2026.1976362 (PMC13668588; doi:10.3389/fpubh.2026.1976362)
Supplement: Supplementary file 1 [file Data_Sheet_1.zip › Table S1. PRISMA_2020 checklist_AP.docx]

| **Section and Topic** | **Item #** | **Checklist item** | **Location where item is reported** |
| --- | --- | --- | --- |
| **TITLE** | | |  |
| Title | 1 | Identify the report as a systematic review. | Section Title:  Human-Centered Industry 5.0 Technologies for Occupational Risk Reduction: A Systematic Review |
| **ABSTRACT** | | |  |
| Abstract | 2 | See the PRISMA 2020 for Abstracts checklist. | Section Abstract |
| **INTRODUCTION** | | |  |
| Rationale | 3 | Describe the rationale for the review in the context of existing knowledge. | Section Introduction |
| Objectives | 4 | Provide an explicit statement of the objective(s) or question(s) the review addresses. | Section Introduction |
| **METHODS** | | |  |
| Eligibility criteria | 5 | Specify the inclusion and exclusion criteria for the review and how studies were grouped for the syntheses. | Section Methods |
| Information sources | 6 | Specify all databases, registers, websites, organisations, reference lists and other sources searched or consulted to identify studies. Specify the date when each source was last searched or consulted. | Section Methods |
| Search strategy | 7 | Present the full search strategies for all databases, registers and websites, including any filters and limits used. | Section Methods |
| Selection process | 8 | Specify the methods used to decide whether a study met the inclusion criteria of the review, including how many reviewers screened each record and each report retrieved, whether they worked independently, and if applicable, details of automation tools used in the process. | Section Methods |
| Data collection process | 9 | Specify the methods used to collect data from reports, including how many reviewers collected data from each report, whether they worked independently, any processes for obtaining or confirming data from study investigators, and if applicable, details of automation tools used in the process. | Section Methods |
| Data items | 10a | List and define all outcomes for which data were sought. Specify whether all results that were compatible with each outcome domain in each study were sought (e.g. for all measures, time points, analyses), and if not, the methods used to decide which results to collect. | Section Methods |
|  | 10b | List and define all other variables for which data were sought (e.g. participant and intervention characteristics, funding sources). Describe any assumptions made about any missing or unclear information. | Section Methods |
| Study risk of bias assessment | 11 | Specify the methods used to assess risk of bias in the included studies, including details of the tool(s) used, how many reviewers assessed each study and whether they worked independently, and if applicable, details of automation tools used in the process. | Section Methods |
| Effect measures | 12 | Specify for each outcome the effect measure(s) (e.g. risk ratio, mean difference) used in the synthesis or presentation of results. | Section Methods |
| Synthesis methods | 13a | Describe the processes used to decide which studies were eligible for each synthesis (e.g. tabulating the study intervention characteristics and comparing against the planned groups for each synthesis (item #5)). | Section Methods |
|  | 13b | Describe any methods required to prepare the data for presentation or synthesis, such as handling of missing summary statistics, or data conversions. | Section Methods  Five domains were considered: (i) selection and representativeness of participants or experimental scenarios, (ii) validity and reliability of technological and occupational risk measurements, (iii) completeness and transparency of experimental procedures, (iv) adequacy of outcome reporting and statistical analysis, and (v) potential sources of confounding, performance, or reporting bias. Each domain was classified as low, some concerns, or high risk of bias based on the methodological information reported in each study (Figure 3) |
|  | 13c | Describe any methods used to tabulate or visually display results of individual studies and syntheses. | Section Methods  The synthesis distinguished technologies primarily supporting risk detection and monitoring from those enabling preventive or corrective interventions. No sensitivity analyses were performed because the review did not involve pooled effect estimates or statistical meta-analysis. |
|  | 13d | Describe any methods used to synthesize results and provide a rationale for the choice(s). If meta-analysis was performed, describe the model(s), method(s) to identify the presence and extent of statistical heterogeneity, and software package(s) used. | Section Methods  The synthesis distinguished technologies primarily supporting risk detection and monitoring from those enabling preventive or corrective interventions. No sensitivity analyses were performed because the review did not involve pooled effect estimates or statistical meta-analysis. |
|  | 13e | Describe any methods used to explore possible causes of heterogeneity among study results (e.g. subgroup analysis, meta-regression). | Section Methods  Given the substantial heterogeneity in technologies, occupational risks, assessment methods, and outcome metrics across the included studies, a quantitative meta-analysis was not conducted. Instead, findings were synthesized narratively and organized according to technological domain, occupational risk addressed, intervention mechanism, and reported worker-related outcomes. |
|  | 13f | Describe any sensitivity analyses conducted to assess robustness of the synthesized results. | Section Methods  The risk of bias of the included studies was assessed independently by two reviewers to determine the extent to which methodological limitations could affect the reliability and interpretation of the reported findings. |
| Reporting bias assessment | 14 | Describe any methods used to assess risk of bias due to missing results in a synthesis (arising from reporting biases). | Section Methods  The risk of bias of the included studies was assessed independently by two reviewers to determine the extent to which methodological limitations could affect the reliability and interpretation of the reported findings. |
| Certainty assessment | 15 | Describe any methods used to assess certainty (or confidence) in the body of evidence for an outcome. | Section Methods  Studies were grouped and tabulated according to technology type, application context, assessment approach, occupational risk, and reported outcomes to identify convergent patterns, quantitative ranges, implementation conditions, and evidence gaps across industrial sectors. |
| **RESULTS** | | |  |
| Study selection | 16a | Describe the results of the search and selection process, from the number of records identified in the search to the number of studies included in the review, ideally using a flow diagram. | Section Methods  The study selection process was conducted according to the Preferred Reporting Items for Systematic Reviews and Meta-Analyses (PRISMA 2020) guidelines. The initial search retrieved 2382 records, comprising 1970 records from Scopus and 412 from Web of Science (WoS). In Scopus, the application of the publication-year filter (2021–2026) reduced the records to 1321, excluding 649 documents; restricting the results to journal articles reduced the dataset to 557 records, excluding a further 764 documents, which constituted the Scopus records entering the identification stage. |
|  | 16b | Cite studies that might appear to meet the inclusion criteria, but which were excluded, and explain why they were excluded. | Section Methods  The screening and eligibility assessment subsequently followed the PRISMA sequence (https://estech.shinyapps.io/prisma_flowdiagram/) (Figure 1). From the 610 unique records entering the screening stage, 472 records were excluded following examination of their titles and abstracts because they did not sufficiently address human-centered ergonomics, Industry 4.0/5.0 technologies, occupational applications, or the defined outcome domains. The remaining 138 records were sought for full-text assessment; 29 records were excluded because the full text could not be retrieved, resulting in 109 articles available for detailed eligibility assessment. Full-text examination led to the exclusion of 31 studies because their findings were predominantly descriptive, they did not provide sufficient experimental procedures or empirical evidence, or their thematic focus was substantially distant from the objectives of the review. |
| Study characteristics | 17 | Cite each included study and present its characteristics. | Section Methods and Section Results |
| Risk of bias in studies | 18 | Present assessments of risk of bias for each included study. | Section Results  Overall, the methodological quality of the included studies varied across the five evaluated domains. The majority of studies exhibited a low risk of bias regarding measurement validity and protocol clarity, driven by the use of validated technological instrumentation and standardized experimental setups. However, high risk of bias and some concerns were frequently observed in participant selection and sample size adequacy, as many experiments were conducted in controlled laboratory settings with limited sample sizes. A detailed breakdown of the risk of bias proportions across all evaluated domains is summarized in Figure 3, while the individual assessment for each of the 78 included studies is presented in Table S2 (Supplementary Materials). |
| Results of individual studies | 19 | For all outcomes, present, for each study: (a) summary statistics for each group (where appropriate) and (b) an effect estimate and its precision (e.g. confidence/credible interval), ideally using structured tables or plots. | Section Results  Scientific evidence published between 2021 and 2026 indicates an evolution from the initial application of collaborative technologies toward more intelligent, adaptive, and continuously monitored human-centred systems for occupational risk assessment. Early research focused on collaborative robots, exoskeletons, augmented reality, digital twins, and sensor-based systems as mechanisms for identifying physical workload, supporting safer task execution, and improving human–technology interaction (40,44,50,51,54,56,61,69,78,87). |
| Results of syntheses | 20a | For each synthesis, briefly summarise the characteristics and risk of bias among contributing studies. | Section Results  Risk of bias and methodological quality assessment (N = 78). Five domains were evaluated: D1 (Selection and scenario representativeness), D2 (Construct validity and measurement reliability), D3 (Ecological setting and shop-floor applicability), D4 (Reporting adequacy and transparency), and D5 (Human factors and confounding control). Overall risk was calculated using the domain-level worst-case rule. The overall high-risk proportion (23.08%, n = 18) matches D3 exactly, as no high-risk scores were observed in Domains 1, 2, 4, or 5. |
|  | 20b | Present results of all statistical syntheses conducted. If meta-analysis was done, present for each the summary estimate and its precision (e.g. confidence/credible interval) and measures of statistical heterogeneity. If comparing groups, describe the direction of the effect. | Section Results  Regarding the evolution of application areas, manufacturing remains the predominant context, particularly assembly operations, where repetitive movements, manual material handling, workstation configuration, and human–robot collaboration generate substantial occupational risk exposure (40,46,49,54–56,65,73,83,93,99,108,110). Automotive manufacturing represents another important application area, with technologies addressing physical fatigue, collaborative robotics, machine vision, and integrated smart manufacturing systems (44,50,52,75,82,115). Textile and apparel production also emerges as a relevant context because repetitive upper-limb movements and constrained postures contribute to persistent musculoskeletal risks, motivating the use of machine vision, machine learning, and automated risk assessment (60,95,107,116). Beyond manufacturing, these technologies have expanded to construction, logistics, agriculture, healthcare, and pharmaceutical environments (45,51,58,70,72,90), demonstrating cross-sector applicability. These applications indicate that human-centred Industry 5.0 technologies can be adapted to diverse work settings where physical workload, repetitive tasks, human–technology interaction, and worker safety represent critical occupational concerns. |
|  | 20c | Present results of all investigations of possible causes of heterogeneity among study results. | Section Results  In this sense, the evolution observed across the studies suggests a transition from technology-assisted ergonomic assessment toward intelligent, predictive, and adaptive approaches to occupational risk management. Early applications primarily demonstrated the feasibility of individual technologies for assessing posture, monitoring physical workload, or supporting safer task execution, whereas more recent studies increasingly combine multiple sensing modalities with artificial intelligence, collaborative robotics, digital twins, and adaptive decision-making (57,62,88,97,104,106). This convergence enables continuous monitoring of worker conditions and supports earlier identification of changes in workload, fatigue, posture, and other occupational risk factors (73,83). Accordingly, the emerging technological paradigm extends beyond the automation of ergonomic assessment toward work environments capable of detecting worker conditions, interpreting individual needs, predicting potential risks, and supporting timely preventive or adaptive modifications to tasks and system configurations. |
|  | 20d | Present results of all sensitivity analyses conducted to assess the robustness of the synthesized results. | Section Results  The cross-sectoral evidence indicates that the transition toward Human-Centred Industry 5.0 is increasingly characterized by moving from isolated risk detection toward integrated and worker-oriented approaches to occupational risk reduction. Across manufacturing, automotive, assembly, logistics, and other industrial environments, computer vision, wearables, digital twins, collaborative robots, exoskeletons, extended reality, and artificial intelligence increasingly combine assessment with prediction, prevention, and adaptive intervention (39,42,59,75,79). This evolution is illustrated by IMU-based assessment, which reduced evaluation time from 70 to 2 min while maintaining comparable RULA classifications (48), and AI-based systems that achieved real-time posture and fatigue recognition with accuracies above 94% in several applications (52,60,94). These findings suggest that digitalization can strengthen occupational risk management by enabling earlier detection and more timely preventive responses. |
| Reporting biases | 21 | Present assessments of risk of bias due to missing results (arising from reporting biases) for each synthesis assessed. | Section Results  However, high risk of bias and some concerns were frequently observed in participant selection and sample size adequacy, as many experiments were conducted in controlled laboratory settings with limited sample sizes. A detailed breakdown of the risk of bias proportions across all evaluated domains is summarized in Figure 3, while the individual assessment for each of the 78 included studies is presented in Table S2 (Supplementary Materials). |
| Certainty of evidence | 22 | Present assessments of certainty (or confidence) in the body of evidence for each outcome assessed. | Section Results  However, high risk of bias and some concerns were frequently observed in participant selection and sample size adequacy, as many experiments were conducted in controlled laboratory settings with limited sample sizes. A detailed breakdown of the risk of bias proportions across all evaluated domains is summarized in Figure 3, while the individual assessment for each of the 78 included studies is presented in Table S2 (Supplementary Materials). |
| **DISCUSSION** | | |  |
| Discussion | 23a | Provide a general interpretation of the results in the context of other evidence. | Section Discussion  The evidence indicates that the effectiveness of Industry 5.0 technologies for occupational risk reduction depends less on technological novelty than on their capacity to transform risk assessment into continuous, objective, and actionable intervention. Computer vision and AI-based systems showed strong performance for postural risk assessment, with recent systems achieving F1 scores above 0.99 and AUC above 0.996 for real-time posture classification (94), while PRoTAS reached an F1 score of 94.52% at 15–20 fps (52). Wearable systems provided complementary advantages by capturing physiological and biomechanical information not reliably inferred from external posture alone. IMU-based assessment reduced evaluation time from 70 to 2 min while maintaining comparable RULA classifications (48), whereas multimodal wearables captured physiological responses associated with exertion and fatigue (45). Thus, computer vision appears particularly suitable for posture and movement monitoring, whereas wearables offer greater value for physiological risk assessment; however, vision remains sensitive to occlusion and environmental conditions, while wearables may affect comfort and worker acceptance. |
|  | 23b | Discuss any limitations of the evidence included in the review. | Section Discussion  The practical implications should address standardized frameworks for integrating ergonomic, technological, and occupational safety indicators within Industry 5.0 systems. Currently, heterogeneous assessment protocols, sensor configurations, artificial intelligence metrics, and outcome criteria are used, limiting direct comparison and transferability between industrial environments (74,77,78). |
|  | 23c | Discuss any limitations of the review processes used. | Section Discussion  Technological fragmentation is another significant gap, as machine vision, wearable devices, digital twins, collaborative robots, and production control systems are frequently evaluated as standalone solutions rather than interoperable components of a unified, human-centered safety architecture (53,81). Therefore, standardized data structures, interoperable interfaces, common validation protocols, and multidimensional occupational risk indicators should be prioritized (41,42). |
|  | 23d | Discuss implications of the results for practice, policy, and future research. | Section Discussion  Future research should move from isolated technological demonstrations toward integrated and predictive human-centred systems for occupational risk prevention. Human Digital Twins represent an important direction because they can combine biomechanical, physiological, cognitive, and contextual information to support dynamic task allocation and fatigue prediction (41,53,59). |
| **OTHER INFORMATION** | | |  |
| Registration and protocol | 24a | Provide registration information for the review, including register name and registration number, or state that the review was not registered. | Section Methods  Not applicable  This study was designed as a cross-sector systematic review and reported in accordance with the 2020 PRISMA (Preferred Reporting Items for Systematic Reviews and Meta-Analyses) statement (35). The review synthesized empirical evidence on human-centered Industry 5.0 technologies that support the assessment, prevention, monitoring, and reduction of occupational hazards in industrial environments related to manufacturing, assembly, logistics, human-robot collaboration, and other sectors. |
|  | 24b | Indicate where the review protocol can be accessed, or state that a protocol was not prepared. | Section Methods  The selection procedure adhered strictly to the four phase PRISMA 2020 protocol: identification, screening, eligibility, and final inclusion <https://estech.shinyapps.io/prisma_flowdiagram/> |
|  | 24c | Describe and explain any amendments to information provided at registration or in the protocol. | No amendments to the registered protocol were mad |
| Support | 25 | Describe sources of financial or non-financial support for the review, and the role of the funders or sponsors in the review. | This research was funded by the Universidad Nacional del Santa under the Call for Competitive Grants for Research Projects Financed with Mining Royalty Resources, Research Groups Category – 2023, pursuant to Resolution No. 749-2023-CU-R-UNS and Contract No. 0022-2025-UNS-OAJ, corresponding to the project “Development of an intelligent biomechanical system in fishing sector workstations to improve job performance". |
| Competing interests | 26 | Declare any competing interests of review authors. | “The authors declare no conflicts of interest.” |
| Availability of data, code and other materials | 27 | Report which of the following are publicly available and where they can be found: template data collection forms; data extracted from included studies; data used for all analyses; analytic code; any other materials used in the review. | "All data generated or analyzed during the study took into account the selected quality procedure, which considered the characteristics of high-impact databases, including the document identifier and its digital registration in the Scopus and Web of Science databases, through access from ([crodriguezy23@ucvvirtual.edu.pe](mailto:crodriguezy23@ucvvirtual.edu.pe) ).” |

*From:*  Page MJ, McKenzie JE, Bossuyt PM, Boutron I, Hoffmann TC, Mulrow CD, et al. The PRISMA 2020 statement: an updated guideline for reporting systematic reviews. BMJ 2021;372:n71. doi: 10.1136/bmj.n71. This work is licensed under CC BY 4.0. To view a copy of this license, visit <https://creativecommons.org/licenses/by/4.0/>
